# Multistage time-to-event models improve survival inference by partitioning mortality processes of tracked organisms

**DOI:** 10.1038/s41598-024-64653-w

**Published:** 2024-06-25

**Authors:** Suresh A. Sethi, Alex L. Koeberle, Anna J. Poulton, Daniel W. Linden, Duane Diefenbach, Frances E. Buderman, Mary Jo Casalena, Kenneth Duren

**Affiliations:** 1 Aquatic Research and Environmental Assessment Center, Department of Earth and Environmental Sciences, Brooklyn College, Brooklyn, NY 11210 USA; 2 Department of Natural Resources and the Environment, Cornell University, Ithaca, NY 14853 USA; 3 Center for Applied Mathematics, Cornell University, Ithaca, NY 14853 USA; 4 Northeast Fisheries Science Center, National Marine Fisheries Service, National Oceanic and Atmospheric Administration, Woods Hole, MA 02543 USA; 5 U.S. Geological Survey, Pennsylvania Cooperative Fish and Wildlife Research Unit, Pennsylvania State University, University Park, PA 16802 USA; 6 Department of Ecosystem Science and Management, Pennsylvania State University, University Park, PA 16802 USA; 7 Pennsylvania Game Commission, Harrisburg, PA 17110 USA

**Keywords:** Ecological modelling, Population dynamics, Statistics

## Abstract

Advances in tagging technologies are expanding opportunities to estimate survival of fish and wildlife populations. Yet, capture and handling effects could impact survival outcomes and bias inference about natural mortality processes. We developed a multistage time-to-event model that can partition the survival process into sequential phases that reflect the tagged animal experience, including handling and release mortality, post-release recovery mortality, and subsequently, natural mortality. We demonstrate performance of multistage survival models through simulation testing and through fish and bird telemetry case studies. Models are implemented in a Bayesian framework and can accommodate left, right, and interval censorship events. Our results indicate that accurate survival estimates can be achieved with reasonable sample sizes ($n\approx 100+)$ and that multimodel inference can inform hypotheses about the configuration and length of survival stages needed to adequately describe mortality processes for tracked specimens. While we focus on survival estimation for tagged fish and wildlife populations, multistage time-to-event models could be used to understand other phenomena of interest such as migration, reproduction, or disease events across a range of taxa including plants and insects.

## Introduction

Advances in tagging and tracking technologies are expanding opportunities to monitor fish and wildlife populations. Among other applications, these tracking approaches can be used to assess the survival of individual study specimens. For example, GPS-enabled tags provide information on the location and activity of tracked specimens that can be used to infer whether and when subjects perish as indicated by the cessation of movements (migratory birds^1^; hares^2^; deer^3^). While applications of GPS-enabled tags have focused on terrestrial specimens, recent studies have demonstrated the utility of popup satellite archival tags for tracking the survival fate of aquatic organisms by monitoring movement accelerations underwater (sharks^4^; cod^5^; halibut^6^). Archival tags are still relatively large and thus must be deployed on larger-bodied animals, but on the other end of the size spectrum, non-archival tags including actively pinging acoustic tags and some GPS-enabled tags have now been miniaturized sufficiently to provide information on specimens on the order of tens of grams in body weight (e.g. juvenile fish^7^; shorebirds^8^).

Observations of the survival fate and timing of mortalities from tracking technologies generate a particularly useful form of information referred to as “time-to-event” data. Time-to-event data can be used to estimate survival functions that provide cumulative survival to any point in time during the observation period^9,10^. Further, because time-to-event models are informed by observations on individually tracked specimens, fates can be linked to subjects’ attributes or behaviour during the study, providing the opportunity to investigate hypotheses about drivers of mortality. By characterizing the time path of mortality, these models provide additional information on survival beyond alternative estimation approaches that collapse time into binary survive-or-not data such as logistic regression. While mortality is a demographic parameter of central interest for fish and wildlife management, the definition of an “event” is general and can encompass other biological processes that could be monitored through in situ observations such as reproductive or migration events.

The combination of tracking technologies and time-to-event statistical models provide a powerful approach for estimating survival for subjects at large, with applications spanning a wide range of taxa including fish^11^, birds^12^, ungulates^13^, and potentially even insects through the use of radio frequency identification chips^14^. However, a challenge common across applications is that the process of catching, handling, and releasing tagged specimens could impact their fates and bias inference about the survival functions associated with “natural” mortality processes^15^. For example, many wildlife species experience capture myopathy whereby the physical stress of being chased and handled can manifest as a post-handling elevated mortality period^16,17^. Similarly, released fish can experience compression barotrauma^18^ as they move from surface waters to pressures at depth, and en-masse releases often attract fish and bird predators that can lead to high initial mortality^19,20^. To address these challenges, time-to-event models that partition survival into discrete stanzas can provide opportunity to isolate mortality sources associated with capture, handling, and release practices from those associated with natural mortality processes.

A small number of existing modelling frameworks have sought to partition time-to-event processes into discrete stages. While these advancements have focused on medical, engineering, or economic applications, they also have use in modelling phenomena in ecological domains. Zero-inflated time-to-event models partition the survival process into two stages represented as a mixture of some probability of experiencing the event of interest immediately upon entry into a study followed by a conventional parametric survival process^21^. In fish and wildlife studies, zero-inflated time-to-event models could be useful for isolating “straight to death” outcomes for captured animals that perish immediately upon release, whereas subjects that survive release can go on to provide information about other mortality processes. Similarly, mixture-cure models partition the survival process into a probability of surviving full term through the end of the study (i.e. “cured” individuals) from subjects who are at risk of experiencing mortality during the study following a conventional parametric survival process^22^. By isolating the cure population, mixture-cure models could provide opportunity to investigate the attributes of subjects that are poised for full term survival versus those that are at risk of mortality hazards throughout the observation period. More recently, models have been developed that combine both zero-inflation and mixture-cure models with a conventional survival model to support the characterization of multiple, potentially overlapping, mortality processes^23,24^.

Time-to-event frameworks that partition survival into distinct phases provide flexibility to assess multiple stages of mortality hazards; however, existing approaches have several limitations. First, zero-inflated or mixture-cure models include immediate death or full-term survival as binary outcomes but fail to provide flexibility in modelling intermediate stages of mortality. Second, the interpretation of mortality hazards estimates from cure models in relation to full term survivors (the cure population) is not clear. For example, mixture-cure survival models imply that some cohort of study subjects are never at risk of death during the study (i.e. the cure population bypasses a given mortality regime)^22^. This assumption may be challenging to meet in field studies as it is unclear how any group of handled, tagged, and released fish or wildlife subjects could not be at risk of mortality from these interventions. Third, existing models that accommodate temporally distinct mortality phases require specifying the timing and length of survival stages a priori or by asserting arbitrary cumulative mortality thresholds that define the length of time over which a given mortality source persists^23^. Instead, models that allow event time data to inform mortality stage structures and stage lengths would provide novel opportunities to learn about survival processes directly from the fates of study subjects. Combined, these limitations suggest a need for time-to-event models that can both represent multiple phases of hazards to isolate mortality sources attributable to study interventions (e.g. capture, handling, and release practices) from those attributable to natural processes, and that can use event times to directly inform the structure and lengths of survival stages.

Here we develop a multistage time-to-event model framework that offers improved inference about the fate of tracked specimens by partitioning survival into sequential stages that reflect key mortality stanzas associated with capture and handling versus those attributable to natural mortality processes. For example, a likely signature for handled and released organisms may include three discrete mortality stages commencing with high immediate mortality upon release, followed by an elevated mortality acclimation period as study subjects recover from stress or injury during handling and tagging, finally followed by a transition to a natural mortality regime for fully recovered individuals. Our approach builds from piece-wise exponential models^10,25^, but further extends these frameworks by allowing the transition times between sequential survival stages to be directly estimated from event time data. We implement the multistage time-to-event model in a Bayesian framework and conduct a combination of simulation testing and case study examples to demonstrate applications. Our multistage survival model framework yields accurate estimates of stage-specific survival rates and can include subject-level covariates. Further, we demonstrate that multimodel selection can inform the number and length of sequential survival stages, providing further insights into the mortality dynamics of tracked organisms.

## Methods

We first describe the multistage time-to-event survival model. Subsequently, we describe Bayesian implementation of the modelling framework and simulation testing. Finally, we describe application of the multistage survival model to a fisheries (cisco, *Coregonus artedi*) and a wildlife (wild turkey, *Meleagris gallopavo*) case study.

### Time-to-event modelling preliminaries

The primary observations used in time-to-event models are “event times” which provide the time, $t$, from the start of a trial ($t=0$) to the occurrence of an event of interest (e.g. death). Event times are non-negative continuous random variables $T\in [0,\infty )$, with density function $f(t)$ and cumulative distribution function $F\left(t\right).$ Time to event survival models are specified through the survival function $S\left(t\right)=\text{Pr}\left(T>t\right)=1-F(t)$, which provides the probability of surviving to time $t$, the hazard function $h\left(t\right)=f(t)/S(t)$, which represents the rate of experiencing the event of interest (e.g. death) at any instantaneous point in time conditional on still being at risk of experiencing the event at that time, and the cumulative hazards function $H\left(t\right)= {\int }_{0}^{t}h\left(u\right)du$ (e.g.^9,25^). The survival function can also be written as: $S\left(t\right)=\text{exp}(-H\left(t\right))$.

A key feature of time-to-event modelling, and one that can bias survival estimation if left unaddressed, is the presence of censorship events whereby the event of interest goes unobserved. Three types of censorship events can occur: right, left, or interval censoring. In fish and wildlife studies, right censoring is most common and occurs when surveillance of tagged specimens ends prior to the event of interest occurring, for example as would be the case if animals survive past the end of monitoring. In left censorship cases, a subject enters a study as having already experienced the event of interest, for example when a tagged animal dies prior to the commencement of monitoring. Finally, interval censoring cases occur when an event of interest transpires during an unmonitored period in a study. Subjects that are observed experiencing the event of interest (i.e. uncensored individuals) contribute to the likelihood through the event time density function, $f\left(t\right)=h\left(t\right)S(t)$. Right censored subjects carry information that an individual survived up to the time of censoring and contribute to the likelihood through the survival function, $S(t)$. Similarly, a left censorship event at time $t$ contributes to the likelihood as $1-S(t)$. Finally, an interval censorship event that occurs between surveillance times ${t}_{1}$ and ${t}_{2}$ with ${t}_{2}>{t}_{1}$ contributes to the likelihood as $S({t}_{1})-S({t}_{2})$.

Let $\delta$, $\omega$, and $\rho$ specify indicator variables for right, left, or interval censorship events, respectively, that take on a value of 1 if a given censorship event has transpired or zero otherwise. Subjects can either experience the event of interest or one of the three censorship events, i.e. $\left(\delta +\omega +\rho \right)\in \{\text{0,1}\}.$ The likelihood for subject $i$’s event time data given a set of parameters specifying the form of the event functions, $\theta$, can be expressed as:

1 $$L\left( {t,t_{1} ,t_{2} {|}\theta } \right) = \left( {f\left( t \right)} \right)^{{\left( {1 - \delta } \right)\left( {1 - \omega } \right)\left( {1 - \rho } \right)}} \times \left( {S\left( t \right)} \right)^{{\left( \delta \right)\left( {1 - \omega } \right)\left( {1 - \rho } \right)}} \times \left( {1 - S\left( t \right)} \right)^{{\left( {1 - \delta } \right)\left( \omega \right)\left( {1 - \rho } \right)}} \times \left( {S\left( {t_{1} } \right) - S\left( {t_{2} } \right)} \right)^{{\left( {1 - \delta } \right)\left( {1 - \omega } \right)\left( \rho \right)}}$$

### The multistage time-to-event survival model

Here, we implement a piecewise hazards function to develop a flexible multistage time-to-event model that can describe tagged animal mortality through distinct survival stages. We define a set of temporal cutpoints, $a$, which partition survival times into $m$ sequential intervals (i.e. stages), $a\in ({a}_{1}=0,\dots ,{a}_{m},{a}_{m+1}=\infty )$, where all cutpoints are non-negative and are ordered such that ${a}_{k+1}>{a}_{k}$ for $k=1,\dots ,m$. Subsequently, we assume that the mortality hazard rate is constant within each stage, although we expect hazards rates to differ across the sequential time stages: ${h}_{k}\left(t\right)={\widetilde{\lambda }}_{k}$ for $k=1,\dots ,m$. Although simple, this step function form of the hazard rate is flexible and can accommodate complicated survival dynamics, as we outline below. With this piecewise specification of constant within-stage baseline hazards, then the cumulative hazards function is the cumulative time passed up to time $t$ multiplied by the respective stage-specific hazard rates. For example, the cumulative hazards for an event time that falls within the first stage is $(t-{a}_{1}){\widetilde{\lambda }}_{1}$, whereas the cumulative hazards for an event time that falls within the second stage is $\left({a}_{2}-{a}_{1}\right){\widetilde{\lambda }}_{1}+(t-{a}_{2}){\widetilde{\lambda }}_{2}$, and so forth.

Finally, in many applications, we seek to model the influence of covariates on survival processes. Our model framework includes covariate effects through a proportional hazards approach, where a baseline hazard rate, $\lambda$, is multiplied by a linear predictor through an exponential function: ${h}_{k}\left(t\right)={\widetilde{\lambda }}_{k}={\lambda }_{k}\text{exp}({{\varvec{x}}} ^{\prime}{\varvec{\beta}})$ where ${\varvec{x}}$ is a vector of covariates with the coefficients ${\varvec{\beta}}$ and the baseline hazard rate $\lambda$ to be estimated. The proportional hazards approach scales the baseline hazard rate according to the effect of covariates; as $\text{exp}({{\varvec{x}}} ^{\prime}{\varvec{\beta}})$ gets large, the hazard rate increases (survival decreases) and vice versa. In the most general form of the multistage model, a common set of covariates could have different stage-specific effects on baseline hazard rates, ${\lambda }_{k}\text{exp}({\varvec{x}} ^{\prime}{{\varvec{\beta}}}_{k})$ (i.e. only coefficients are indexed by $k$), or different covariates could be specified for different survival stages, ${\lambda }_{k}\text{exp}({{\varvec{x}}}_{k} ^{\prime}{{\varvec{\beta}}}_{k})$. Note that intercept terms should not be included in the linear predictor as this would equate to estimating two baseline hazard rates that are multiplicative and thus confounded.

Typically, time interval cutpoints in piecewise hazards models are asserted by the user^10,25,26^; however, in describing tagged animal survival dynamics, it may be useful to specify cutpoints as estimated parameters that are directly informed from the data. In this case, care need be taken to ensure estimated cutpoints are appropriately ordered, for example by parameterizing cutpoints with additive shifts representing the time spent in a given interval, ${\widetilde{\tau }}_{k}$, that are estimated, e.g. ${a}_{k+1}={a}_{k}+{\widetilde{\tau }}_{k}$. Alternatively, informed priors in a Bayesian model implementation that ensure estimated cutpoints follow a sequential order could be attempted. As with covariate effects on the baseline hazard rates, it is possible to include covariates effects on estimated interval cutpoints, allowing the length of time spent in survival stages to vary across subjects. For example, in tagged animal studies, we may suspect that specimen size affects both survival and the time spent in post-release acclimation stages. We implement a proportional effects form to include covariate effects on the time spent in a given interval, whereby ${\widetilde{\tau }}_{k}={\tau }_{k}\text{exp}({\varvec{z}} ^{\prime}{\varvec{\zeta}})$ where ${\varvec{z}}$ is a vector of covariates; coefficients ${\varvec{\zeta}}$ and the baseline time spent in the stage, ${\tau }_{k}$, are to be estimated.

To assemble the full likelihood, we specify the hazard rate and associated survival functions, incorporating covariate effects on stage-specific hazard rates and any estimated interval cut points:

2 $$h\left( {t_{i} } \right) = {\text{ exp}}\left( {{\varvec{x}}_{{k^{*} \left( {t_{i} } \right),i}} ^{\prime}{\varvec{\beta}}_{{k^{*} \left( {t_{i} } \right)}} } \right) \lambda_{{k^{*} \left( {t_{i} } \right)}} { }$$

3 $$S\left( {t_{i} } \right) = \exp \left( { - H\left( {t_{i} } \right)} \right) = {\text{exp}}\left( { - \mathop \sum \limits_{k = 1}^{m} \exp \left( {{\varvec{x}}_{k,i} ^{\prime}{\varvec{\beta}}_{k} } \right) \lambda_{k} R\left[ {i,k} \right]} \right)$$

where ${k}^{*}({t}_{i})$ is a function returning an integer indicating in which stage that individual $i$’s event time falls (i.e. if ${t}_{i}\in [{a}_{i,k},{a}_{i,k+1})$ then ${k}^{*}\left({t}_{i}\right)=k$). $R[i,k]$ provides the times within stages needed to sum the cumulative hazards:

$$R\left[ {i,k} \right] = \left\{ {\begin{array}{*{20}c} {\tilde{a}_{i,k + 1} - \tilde{a}_{i,k} } & {{\text{if}}} & {t_{i} > \tilde{a}_{i,k + 1} } \\ {t_{i} - \tilde{a}_{i,k} } & {{\text{if}}} & {t_{i} \in \left( {\tilde{a}_{i,k} ,\tilde{a}_{i,k + 1} } \right]} \\ 0 & {{\text{if}}} & {t_{i} \le \tilde{a}_{i,k} } \\ \end{array} } \right.$$

where $${\widetilde{a}}_{i,k+1}=\left\{\begin{array}{cc}{\widetilde{a}}_{i,k}+{\tau }_{k}\text{exp}({{\varvec{z}}}_{i,k} ^{\prime}{{\varvec{\zeta}}}_{k})& \text{if interval cutpoint }k+1 \text{ is estimated }\\ {a}_{k+1}& \text{if interval cutpoint }k+1 \text{ is asserted}\end{array}\right.$$ and ${\widetilde{a}}_{i,1}=0$ (i.e. the first interval cut point value is always set at time = 0). Model components from Eqs. (2), (3) are substituted into the likelihood from Eq. (1) to provide the full likelihood as $L(D| \theta )={\prod }_{i=1}^{n}{L}_{i}({D}_{i}| \theta )$ where ${D}_{i}({{\varvec{y}}}_{i},{{\varvec{I}}}_{i},{{\varvec{x}}}_{i})$ represents the event times (either a single observed event time, a single left or right censor time, or a pair of flanking interval censorship event times), indicator variables that code censorship events, and the covariate vector for subject $i$, respectively, and $\theta$ is the set of estimated and specified model parameters.

The multistage time-to-event framework provides substantial flexibility in describing event processes for fish and wildlife applications. We suggest that a 3-stage form of the multistage survival model may be a useful general structure to characterize the sequential release, acclimation, and natural mortality periods for many tagged animal studies. In the first stage, animals could perish immediately upon release. These “straight to death” outcomes are a form of zero inflation in survival trials and we incorporate this in the multistage model as an initial time interval of short length, for example by specifying a unit time interval for this stage by setting ${a}_{1}=0$ and ${a}_{2}=1$. Longer straight to death time periods could be specified, but we note that a unit time interval stage is equivalent to specifying a binomial mortality model with success probability equivalent to ${\widetilde{\lambda }}_{1}$ (but see Text S4). Subsequently, animals that survive initial release mortality transition to an “acclimation” period whereby mortality may be elevated above a “natural mortality” rate. To provide flexibility in describing tagged animals’ survival experiences, we can specify the time spent in the acclimation interval, ${\widetilde{\tau }}_{2}$, and thus the exit time from this period as ${a}_{3}={a}_{2}+{\widetilde{\tau }}_{2}$, as an estimated parameter, potentially including covariate effects: ${\widetilde{\tau }}_{2}={\tau }_{2}\text{exp}({\varvec{z}} ^{\prime}{\varvec{\zeta}})$. After the acclimation interval, animals transition to a natural mortality regime.

Other relevant model forms for fish and wildlife applications can easily be specified. For example, a simple zero-inflated survival model could be generated by specifying two stages: a straight to death mortality stage followed by a post-release survival stage. Similarly, a conventional piecewise exponential model can be implemented by asserting all interval cutpoints exogenously as opposed to estimating them from observed event time data (e.g.^27^). Further, depending on analysts’ assumptions about the processes driving mortality, multistage models could be used to represent overlapping cause-specific hazards by parameterizing stage specific mortalities as sums of different hazards. For example, overlapping mortality processes could be specified for capture and handling hazards (${\lambda }_{C}$), acclimation mortality (${\lambda }_{A}$), and natural mortality (${\lambda }_{N}$), whereby stage 1 hazards may include all three mortality sources (${\lambda }_{1}={\lambda }_{C}+{\lambda }_{A}+{\lambda }_{N}$), stage 2 only acclimation and natural mortality (${{\lambda }_{2}=\lambda }_{A}+{\lambda }_{N}$), and finally with stage 3 hazards encompassing only natural mortality (${\lambda }_{3}={\lambda }_{N})$. Such specifications could be useful for estimating the magnitudes of different mortality processes.

### Bayesian implementation of multistage survival models

We implemented multistage survival models in a Bayesian framework using JAGS^28^ as implemented in R version 4.3.1 with the *rjags* package^28–30^. We use the “zeros trick” to compute the multistage time-to-event likelihood within the JAGS Markov Chain Monte Carlo (MCMC) sampler^25,26,31^ (see Text S4). Vague uniform priors were specified for all estimated parameters as detailed in model code provided in the supplementary materials. Common alternative priors include diffuse gamma priors for hazard rates and normal priors for linear predictor coefficients; however, exploratory testing indicated similar model fit results with uniform priors versus gamma and normal priors.

Models were implemented with three MCMC chains each with 1000, 5000, and 5000 iterations for the adaptation, burn in, and posterior sampling phases at a thin rate of 20. With this configuration, we found that the largest models (3-stages, covariate effects, $n$ = 1000) took ≈ 30 min to complete on a laptop computer with an Intel i9-11950H processor with 2.60 GHz base frequency and 32.0 GB of RAM. Chains were initiated with best guess parameter values with a small amount of random variation asserted for repeated model implementations. We assessed model convergence by ensuring the Gelman–Brooks–Rubin $\widehat{R}$ convergence statistics^32^ were < 1.1 for fitted parameters. Annotated JAGS code for a suite of 3-, 2-, and 1-stage time-to-event models with and without covariate effects and estimated stage transitions are presented in the supplementary materials (Text S1–S5). While not exhaustive, we hope these models provide sufficient examples such that users can customize multistage time-to-event models to their specific applications of interest.

### Simulation analyses

We implemented two tests to assess the performance of multistage time-to-event models. First, we explored parameter estimation accuracy across data sets simulated from a 3-stage survival process with covariate effects on hazard rates and on the time spent in the second, i.e. “acclimation,” stage. Covariate values could be viewed as representing specimen size data and ranged from unitless values of 0.0 to 100.0. Different covariate effects were specified for each respective survival stage and the estimated transition time. The simulated monitoring period, stage lengths, and survival parameters were tuned to reflect plausible conditions for ecological studies (Text S1). Size effects produce a ≈ 50% increase in stage-specific survival and a ≈ 50% decrease in the time spent in the acclimation stage from the smallest to largest simulated subject sizes (Fig. S1). Event times were generated following stage-specific exponential time-to-event processes using the simsurv R package^33^ (Text S1). Subjects that expired in the first survival stage were coded as left censorship events with censor time equal to the end of the first stage (see Text S4). Subjects that survived full term were coded with a right censorship time equal to the terminal monitoring time. For each sample size *n* = 100, 200, 300, 500, and 1000, we simulated 50 data sets and fit a multistage survival model structured that matched the data generating process, summarizing bias and precision outcomes for estimated parameters (hazard rates, transition times, and “size” covariate coefficients). Subsequently, a similar simulation-estimation protocol was implemented without covariate effects on hazard rates or transition times. Finally, we conducted an additional set of simulations to assess parameter estimation accuracy in the presence of interval censorship. Using the 3-stage model structure without covariate effects, we asserted an interval censorship window of 25 days in the second “acclimation” stage and a 100 days window in the third “natural mortality” stage. We randomly chose subjects to expose to potential interval censorship, with a target censorship rate of 10% of individuals.

Second, we implemented a multimodel inference test to assess ability to discriminate between competing model structures. We simulated data from 3-, 2-, and 1-stage survival processes, exploring models with and without covariate effects on hazard rates, and if present, transition times out of the second survival stage. The 2-stage model represents a zero-inflated exponential survival model, whereas the 1-stage model is equivalent to a conventional exponential parametric survival model. We simulated 50 data sets from each model structure and then challenged each data set with the suite of six candidate models (3-, 2-, or 1- stage, with or without covariate effects). Fitted model support was judged using the deviance information criterion (DIC) using the ${\text{pV}}$ model complexity penalty term^34^. Subsequently, we tallied the proportion of trials for which the correct model structure was identified as the top DIC model or was within 2.0 DIC units of the top DIC model, i.e. indicating that the correct model structure received comparable support to the top DIC model.

### Case studies

In the fisheries case study, we assessed survival of stocked juvenile cisco in a species reintroduction effort in Keuka Lake, New York, U.S.A. with data on *n* = 209 tagged fish provided upon request by the New York State Department of Environmental Conservation and described in Koeberle et al.^35^. Cisco were extirpated from Keuka Lake by the early 1990s and managers are currently attempting to restore the population through hatchery stocking. Our analysis goal was to characterize the different stages and intensity of mortality for stocked cisco to inform fish release practices. From 2019–2021, juvenile cisco were surgically implanted with small acoustic tags (Lotek JSAT tags, Newmarket, Ontario, Canada)^7^ to track their post-release survival across a whole-lake acoustic telemetry array. Death events were specified as the date after which a given subject’s tag was no longer detected on the receiver array. Juvenile cisco were released in annual cohorts at different ages (smaller 10 month old “age-0” fish, or larger 18–22 months old “age-1” fish). We implemented a suite of 1-, 2-, and 3-stage survival models, exploring support for age or size impacts on survival with DIC-based model selection. We specified the initial straight to death mortality period as a unit time interval of 1 day reflecting the 24 h period immediately following fish release.

In the wildlife case study, we estimated survival of a wild turkey population as part of a larger movement study conducted over 2022–2023 in Pennsylvania, U.S.A. We used data on* n* = 358 specimens fitted with backpack-style global positioning system-ultra high frequency (GPS-UHF) tags (70 g, not including the harness; 6400 mAh battery; e-obs GmbH, Gruenwald, Germany). Wild turkey sampling was conducted under Pennsylvania State University IACUC Protocol # PROTO202202180 and methods are reported in accordance with animal research: reporting of in vivo experiments (ARRIVE) guidelines. Wild turkey were captured using rocket nets baited with corn and fitted with a GPS tag^36^. Wild turkey can experience post-release mortality due to capture and tagging stress and typically mortalities that occur < 14 days after release are excluded from further analysis (e.g.^37^.). Our goal in this case study was to assess the intensity and timing of potential capture-related mortality, focusing on a 90 days post-release period. We implemented a suite of 1-, 2-, and 3-stage survival models, exploring support for existence of an elevated “capture effects” mortality stage. We also explored support for release weight effects on stage-specific survivals.

Case study models were specified with vague uniform priors on fitted parameters (Text S2, S3). Continuous covariates were mean-centred to improve parameter identifiability. Model convergence was considered complete when all fitted parameters had $\widehat{R}<1.1$, implementing additional rounds of posterior sampling when necessary. The most complicated case study models (3-stages, covariates on survivals and transition times) took ≈ 10 min to complete. Top DIC-supported multistage models were plotted against Kaplan–Meier curves to evaluate model fit against an empirical description of survival.

## Results

### Simulation testing

**Figure 1:**
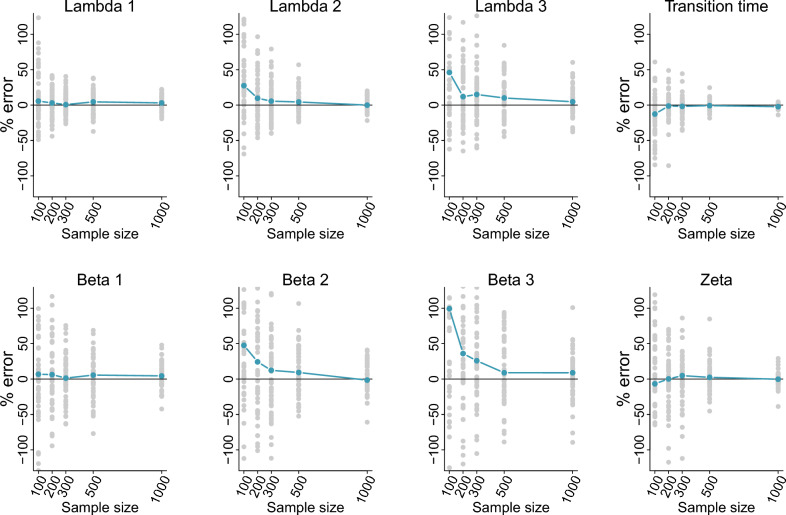
Bias simulation testing results for a three-stage time-to-event survival model including simulated covariate effects on stage-specific hazard rates and on the time spent in the second survival stage. Gray points indicate percent error (100*(estimated parameter value − true parameter value)/true parameter value) results for each respective simulation iteration (50 data sets simulated for each sample size). Blue lines indicate median percent error values across simulations. Lambda’s represent stage-specific baseline hazard rates, “transition time” is the baseline time spent in the second survival stage, beta’s are coefficients on the linear predictor scale for covariate effects on hazard rates, and zeta is the coefficient on the linear predictor scale for covariate effects on the time in stage two.

Simulation testing with 3-stage models that included stage-specific “specimen size” covariate effects on hazard rates and transition times indicates multistage time-to-event models produce accurate parameter estimates that converge to their true underlying values as sample sizes increased (i.e. statistical consistency; Fig. 1). Baseline hazard rate estimates and covariate coefficient estimates approached zero mean bias for samples sizes on the order of *n* = 200 to 300 or larger. Further, the freely estimated transition times out of the second “acclimation” survival stage were accurately estimated across all simulated sample sizes. Models without covariate effects on stage-specific survivals or survival stage transition times demonstrated low bias across all sample sizes simulated (Fig. S2). Similarly, baseline hazard rates and transition times were estimated accurately across all sample sizes tested when interval censorship events were included (Fig. S3). Because baseline simulation scenarios already contained left censorship events in the form of stage-1 deaths and terminal right censorship events for full term survival subjects, these results indicate that multistage time-to-event models can achieve accurate parameter estimation under all three censorship event types. Finally, multistage models achieved good parameter estimate precision (Fig. S4). In simulated 3-stage models with covariate effects on hazard rates and transition times, parameter estimates achieved coefficients of variation (i.e. 100 $\times$ standard deviation of posterior samples/mean of posterior samples) of ≈ 50% or lower for sample sizes of 100–200. Similarly, baseline hazard rates and transition time estimates for 3-stage models without covariate effects achieved estimate parameter coefficients of variation on the order of 10–30% at the smallest sample tested of *n* = 100.

Information Criterion-based model inference testing correctly identified the true data generating process among candidate multistage model structures with high accuracy, particularly in cases with *n* > 100 samples (Table 1). Simulated data generating processes without covariate effects were correctly identified with 89% accuracy or higher even at relatively low samples sizes of *n* = 100. More complicated data generating processes with specimen “size” effects were more difficult to identify at lower sample sizes; however, at sample sizes of 250 or larger, model selection accuracy exceeded 90%.

**Table 1:** Deviance information criterion model selection testing results for multistage time-to-event survival models across simulated data generating processes and sample sizes.

True model structure^+^	True model structure within 2.0 DIC units of the top DIC model (% of trials)		
	*n* = 100; (%)	*n* = 250; (%)	*n* = 500; (%)
3-stage, with covariates	43	90	100
3-stage, no covariates	97	94	97
2-stage, with covariates	78	98	99
2-stage, no covariates	92	92	90
1-stage, with covariates	80	91	98
1-stage, no covariates	89	94	97

^+^Models with covariates include a simulated “specimen size” effect on stage-specific survival rates, and for 3-stage models, a size effect on the time spent in the second survival period.

### Cisco case study

**Figure 2:**
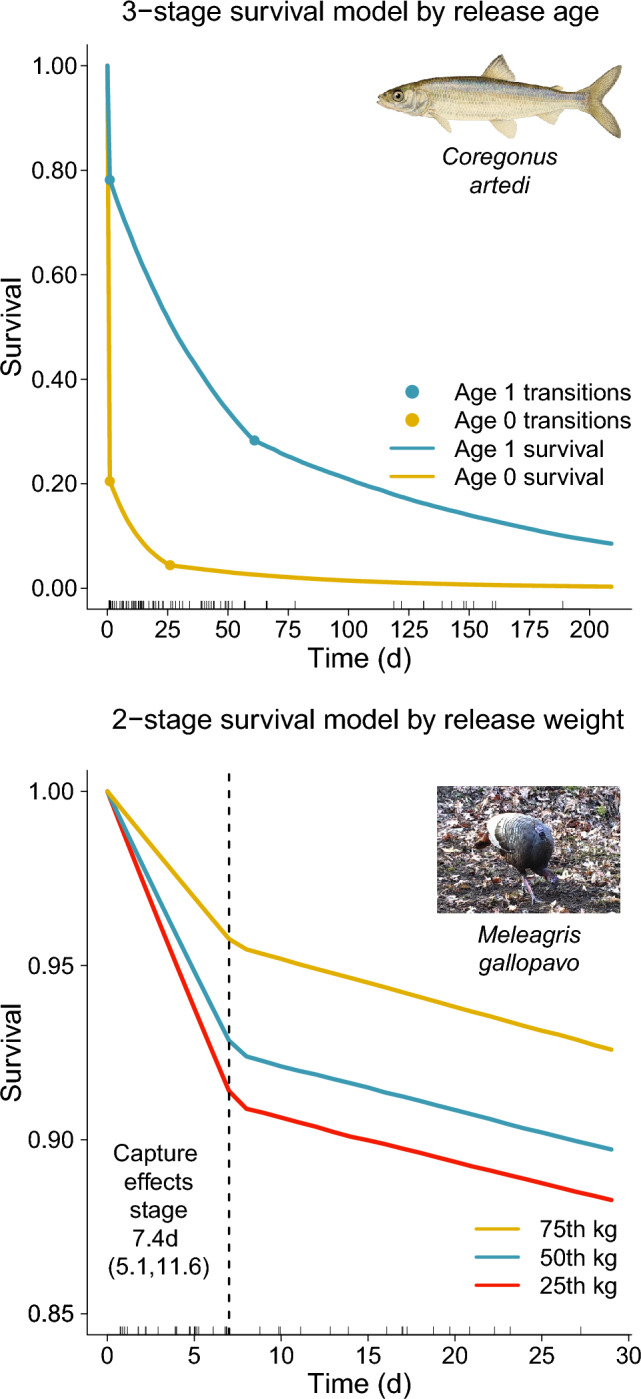
Survival plots for best deviance information criterion-supported multistage time-to-event models fit to cisco (top panel; *Coregonus artedi*) and wild turkey (bottom panel; *Meleagris gallopavo*) case study data. Cisco data were best described by a 3-stage model with a straight to death mortality stage upon fish release, followed by an elevated mortality acclimation stage, and finally a lower long term natural mortality regime. Cisco age-at-release was supported as affecting stage-specific survival and the transition time out of the acclimation stage (release age-0 transition time with 95% credibility intervals: 24.9 days (20.6, 32.4 days); age-1: 59.4 days (47.3, 98.8 days)). Age-1 fish had substantially lower mortality across all survival stages. Turkey data were best described by a 2-stage model with a post-release elevated mortality period of 7.4 days (95% credibility interval: (5.1, 11.6 days)) followed by a lower long term mortality regime. Turkey release weight was supported as affecting survival during the initial post-release effects period, whereby larger birds experienced lower mortality attributable to capture and handling effects (survival by release weight quantiles shown). Rugs show mortality events. Inset photos credits: the cisco picture is by E. Edmonson and is freely available for use in the public domain at: https://commons.wikimedia.org/wiki/File:Cisco.jpg; the wild turkey photo is by M.J. Casalena and is used with permission from author.

All models in the DIC 95% confidence set indicated support for three distinct survival stages and support for age at release as being associated with survival for introduced juvenile cisco in Keuka Lake (Table 2). The top-DIC model, which accounted for 71% of relative DIC weight, included fish release age effects on all three stage survivals and the time spent in the second “acclimation” stage. Survival estimates from this model corresponded closely with empirical Kaplan–Meier summaries, indicating a good fit to the observed cisco survival times (Fig. S5). Estimates for best supported multistage model suggest age-1 fish survived better across all three survival stages compared to age-0 released fish, although age-1 fish spent more days in the second “acclimation” stage (Fig. 2; Table 3). Age-1 fish are typically larger sized than age-0 fish; however, models with a fish length covariate in lieu of fish age were not well supported. We suspect this may be related to a size threshold associated with age, whereby fish released at age 1 are above a minimum size threshold to avoid predation or energy depletion. Survival estimates from the best DIC-supported model indicate that substantial mortality occurs immediately upon release and during a relatively short acclimation phase before both age cohorts settle on a lower long term survival regime (Table 3).

**Table 2:** Cisco (*Coregonus artedi*) and wild turkey (*Meleagris gallopavo*) case study Deviance information criterion model selection results^+^.

Case study	Model structure	DIC	Relative DIC weight	Cumulative DIC weight
Cisco	3 stages, Age effects on all survival rates and on transition time out of the 2^nd^ ‘acclimation’ stage	1140.4	0.707	0.707
	3 stages, Age effects only on stage 1 & 2 survival rates	1142.8	0.211	0.919
	3 stages, Age effects on all survival rates but not on transition time out of the 2^nd^ ‘acclimation’ stage	1144.9	0.075	0.994
Turkey	2 stages, Release Weight effects on both stage 1 and 2 survival	839.2	0.441	0.441
	2 stages, Release Weight effects on stage 1 survival only	840.2	0.261	0.702
	2 stages, Release Weight effects on both stage 1 and 2 survival and on the transition time out of the 1^st^ ‘acclimation’ stage	840.8	0.194	0.896
	2 stages, Release Weight effects on stage 1 survival only	842.8	0.075	0.971

^+^Results are shown for 95% model confidence sets.

**Table 3:** Fitted and derived parameters from the best deviance information criterion supported cisco (*Coregonus artedi*) and wild turkey (*Meleagris gallopavo*) case study models^+^.

Case study	Parameter	Group	Posterior median	95% credibility interval
cisco	Time in “acclimation” stage 2	Age-0	24.8 days	(20.6 days, 32.4 days)
		Age-1	59.4 days	(47.3 days, 98.8 days)
	Survival through stage 1	Age-0	20.5%	(14.4%, 26.3%)
		Age-1	78.2%	(71.3%, 86.0%)
	Survival through first 45 days	Age-0	3.2%	(1.3%, 5.7%)
		Age-1	35.9%	(26.1%, 43.6%)
	Equivalent annual survival^†^ stage 3	Age-0	0.5%	(< 0.1%, 5.1%)
		Age-1	5.5%	(0.2%, 12.8%)
Turkey	Time in “acclimation” stage 1	All subjects	7.4 days	(5.1 days, 11.6 days)
	Survival through stage 1	25th weight percentile	90.3%	(85.5%, 93.9%)
		50th weight percentile	91.9%	(88.3%, 94.9%)
		75th weight percentile	95.0%	(91.7%, 97.5%)

^+^All survival values are translated into discrete time estimates and reported as a percentage, e.g. discrete annual survival, $\widetilde{S}$, given a hazard rate $\lambda$ is $\widetilde{S}={100\times e}^{-365\lambda }$. Model structures: turkey: 2 stages, release weight effects on stage 1 survival only; cisco: 3 stages, age-at-release effects on all survival rates and on the time spent in the second, i.e. “acclimation,” stage.

^†^Calculated as the discrete annual survival equivalent at a full year of the stage 3 hazard rate.

### Turkey case study

All models in the DIC 95% confidence set indicated support for two distinct survival stages and support for release weight as being associated with turkey survival (Table 2). The top three DIC-supported models all received comparable support and thus we chose the most parsimonious model of two-survival stages with release weight effects on stage one survival only as the best DIC-supported model; however, in all cases where release weight was included as a covariate on stage-specific survival, coefficient estimates indicated that larger turkeys survived better than smaller turkeys. Results from the best-supported DIC model indicate the length of the initial post-release elevated mortality period for tagged turkeys was 7.0 days (95% CrI 5, 10.8 days; Table 3). The mortality baseline hazard rate was 6.0 times (95% CrI 3.0, 10.6) greater during this initial post-release phase compared to the subsequent survival stage where birds had presumably recovered from initial handling and capture effects (Fig. 2). This best-supported DIC model also demonstrated a close correspondence to Kaplan–Meier summaries of turkey survival times (Fig. S5). Multimodel selection indicated a lack of support for the addition of an initial straight to death stage (i.e. a 3-stage model).

## Discussion

Multistage models express sequential survival regimes and provide opportunity to partition mortality processes into those associated with specimen capture and handling from those related to natural mortality. Models performed well in simulation trials, demonstrating that accurate and precise parameter estimates related to survival processes could be achieved at sample sizes commonly achievable in fish and wildlife studies. Further, while multistage models can represent complex survival dynamics, DIC-based model selection showed good performance in identifying underlying survival process structures in terms of both the influence of individual-level covariates and the number and length of sequential survival stages.

Two important benefits of multistage time-to-event survival models are apparent. First, if it can be assumed that handled specimens fully recover and acclimate to their environment at large after some point, then multistage models provide opportunity to characterize natural mortality rates as uncontaminated from preceding capture and release-based mortality processes. For example, our analyses of cisco and wild turkey case studies showed evidence of high initial mortality stages influenced by handling and release interventions. Subsequently, estimated survival paths showed a clear transition to a lower long term mortality regime that may be representative of natural mortality (Fig. 2). Thus, assessment of mortality drivers and survival estimates from the terminal sequential survival stage could be used to inform natural mortality dynamics in isolation from initial impacts associated with study interventions.

Second, partitioning survival into discrete stages reflective of study intervention versus ecological processes provides opportunity to learn about the impacts of capture and handling. These insights can then be used to identify actions to address mortality drivers. In the cisco case study, the goal of the conservation effort is to reestablish an extirpated native species to improve the food web resilience of Keuka Lake, New York, U.S.A., for which the primary management tool available is stocking with hatchery-reared juvenile fish. Multistage survival models indicated substantial “straight to death” mortality immediately upon release followed by several weeks of elevated mortality following release during a putative “acclimation” stage. Further, model selection indicated support for age effects where the larger age-1 fish survived substantially better through both of these high mortality stages (Table 3). Thus, multistage modelling results suggest that growing out older specimens to stock and simple changes to release practices such as acclimating fish in pens in situ for a brief period of time (so called net pen stocking, e.g. Connerton et al.^38^) could reduce mortality and improve chances for a successful species reestablishment. Similarly, the turkey case study data show clear adverse effects associated with capture, handling, and tagging during a relatively brief post-release survival window. Whereas many wildlife survival modelling studies employ ad hoc methods to purge out potential capture related deaths by dropping records for subjects that expire during an asserted post-release time window^39,40^, multistage models utilize all available records and can provide novel insights by generating direct estimates of the intensity and length of a capture impacts stage (Fig. 2).

Multistage time-to-event models provide considerable flexibility in describing how mortality processes may change over time. Here, we explored 1-, 2-, and 3-stage survival processes, but more elaborate survival stage structures such as seasonal mortality regimes could also be implemented for longer monitoring studies. Alternatively, models can be specified with many sequential intervals along a regularly spaced time grid to reflect a conventional piecewise exponential survival model^10,27^. That said, generic piecewise models may miss inferential opportunity as there can be insights to be gained by directly testing survival stage structures that reflect hypotheses regarding biological or environmental processes. We caution, however, that additional model complexity involves a higher burden for parameter estimation and thus increased data needs. One potential shortcoming of multistage modelling is the possibility of developing overly complicated models that surpass the information available in tracked specimen data. Fortunately, our simulations suggest that with adequate sample sizes, information theoretic based model selection holds promise for identifying parsimonious multistage model structures. Strategic testing of both complex and reduced multistage models—including conventional single-stage parametric models—can then be conducted to identify model structures with a minimum amount of complexity necessary to describe a given data generating process.

As technologies to monitor the fate of specimens at large continue to advance, we anticipate expanding scope for time-to-event survival models. The multistage models and data set sizes explored here (up to 3 stages and up to *n* = 1000 data) converged relatively quickly using JAGS MCMC samplers. More complex models or larger data sets may take long run times. Thus, improvements in faster samplers may help make multistage time-to-event models more user friendly^41^. Here, we focus applications of multistage models on fish and wildlife survival estimation; however, the scope for multistage models is broader and could encompass a range of biological phenomena of interest that can be modelled with time-to-event approaches including migration strategies^42,43^, reproduction events^44,45^, or wildlife disease outcomes^46,47^, among others.

### Supplementary Information

Supplementary Information 1.

Supplementary Information 2.

Supplementary Information 3.

## Data Availability

Data for case studies are available as Supplementary Information files published with this article. R and JAGS code to implement multistage survival models is provided in Supplemental Texts S1–S5.
