# Supplementary material for: Multistage time-to-event models improve survival inference by partitioning mortality processes of tracked organisms
Source: Sci Rep. 2024 Jun 25;14:14628. doi: 10.1038/s41598-024-64653-w (PMC11199507; doi:10.1038/s41598-024-64653-w)
Supplement: Supplementary file 2 — Supplementary Information 2. [file 41598_2024_64653_MOESM2_ESM.html]

SupplemenatryMaterials.knit


## **Multistage time-to-event models improve survival inference by partitioning mortality processes of tracked organisms**

Supplementary materials   
 v. May 18, 2024

**Contents**   
 **Text S1: Multistage
time-to-event data simulation and model code vignette**
  
  **Text S2: Multistage
time-to-event cisco *(Coregonus artedi)* case study
vignette**   
 
**Text S3: Multistage time-to-event wild turkey case study
*(Meleagris gallopavo)* vignette**   
  **Text S4: Important points for fitting
multistage time-to-event models**   
  **Text S5: Multistage time-to-event
model scripts**   
 
**Figure S1: Simulated “specimen size” covariate effects on
survival and stage transition times for a 3-stage survival
process**   
 
**Figure S2: Parameter estimation bias testing for a 3-stage
time-to-event survival model without covariate effects**
  
  **Figure S3: Parameter
estimation bias testing for a 3-stage time-to-event survival model
without covariate effects and with the inclusion of interval censorship
events**   
 
**Figure S4: Parameter estimation precision testing for a 3-stage
time-to-event survival models**   
 **Figure S5: Comparison of best
DIC-suppored case study model fits against Kaplan-Meier
curves**

### **Text S1: Multistage time-to-event data simulation and model code vignette**

Time-to-event data for model testing in the main text were simulated
under multiple data generating processes that define the number of
survival stages, covariate relationships, and survival rates. In the
most complex data generating process we created, we simulated a 3-stage
time-to-event survival process with a short 1 time unit initial
“straight to death” stage, followed by a 50 time unit second
“acclimation” stage, and a 315 time unit third stage for a ~365 time
unit total observation period. A simulated “specimen size” covariate
effect was included, whereby larger subjects have greater survival and
lower time in the acclimation period relative to smaller sized subjects
(Figure S1). Below we provide code for the data simulation module, and
code to package data and fit a JAGS model with a structure that matches
the simulation data generating process. Model scripts are provided in
**Text S5**.

```
# setwd("Your working directory here") # Set the directory to the location where JAGS model scripts are stored.
library(simsurv) # For simulating exponential data
library(rjags) # For fitting JAGS models

# User specifies-------------------------------------------------------------- 
# Simulation specifications
  n. <- 300 # Sample size to simulate
# Stage lengths: 3 stages w/ first survival stage equivalent to a 'unit' time interval, here simulating e.g. 
# a 366 d total observation period
  tau1 <- 1 # First interval length in time currency (e.g. days)
  tau2 <- 50 # Second interval length
  tau3 <- 315 # Third interval length
# Baseline survival rates, here parameterized as discrete rates over a given stage, translated 
# to instantaneous hazard rates below
  S1 <- 0.5 # Proportion of subjects that survive stage 1
  S2 <- 0.5 # Proportion of subjects that survive stage 2
  S3 <- 0.7 # Proportion of subjects that survive stage 3
# Covariate effects on baseline hazard rates and on the baseline time spent in the  
# 'acclimation' period (stage 2)
  # Mock specimen 'size' continuous covariate values
    size.v <- sample(seq(0,100),n.,replace=T)
  # Coefficient values; with survivals above, these generate approximately a 50% increase in
  # survival from size 0 to size 100, or a halving in the time spent in the 'acclimation' stage.
    beta_1 <- -0.01 # Coefficient for effect of 'size' on baseline hazard for stage 1 
                    # (negative value => higher survival for larger specimens)
    beta_2 <- -0.01 # Coefficient for effect of 'size' on baseline hazard for stage 2
    beta_3 <- -0.01 # Coefficient for effect of 'size' on baseline hazard for stage 3
    zeta <- -0.007  # Coefficient for effect of 'size' on baseline time in stage 2 
                    # (negative coefficient implies less time in stage 2)
# End user specifies--------------------------------------------------------------
```

Simulate survival times and encode censorship events as needed:

```
# Package and process the simulation parameters specified above
  # Define stage start and end times; for m stages, there are m+1 endpoints starting from time = 0
    a.v <-  c(0, tau1, tau1+tau2, tau1+tau2+tau3)                 
  # Define a helper function to map discrete stage-specific survivals (S) to 
  # instantaneous hazard rates given a period of T time units (e.g. days)
    dis_to_instant <- function(S,T){ (-1*log(S))/T }
  # Baseline instantaneous hazard rates
    lambda.v <- c(dis_to_instant(S1,a.v[2]-a.v[1]), 
                  dis_to_instant(S2,a.v[3]-a.v[2]), 
                  dis_to_instant(S3,a.v[4]-a.v[3]))  
# Create a storage object for simulated data; this will match data structures for case study data as well.
# Include censorship indicator variable placeholders (Delta: right censorship, Omega: left, Rho: interval).
# 1 indicates a given censorship event occurred, 0 otherwise.
# 'Time' is used for observed events, right censorship times, and and the start time of an interval 
# censorship event window.
# 'Time2' is used exclusively for the end time for an interval censorship event window. 
# Simulations below can include left (death in stage 1) or right censorship (subjects surviving full term), but 
# interval censorship events are not simulated here.
  data.df <- data.frame(Subject = 1:n., Time=NA, Time_2=0, Delta=0, Omega=0, Rho=0, Size=size.v)
# Simulate subject-level data
  for(i in 1:n.){
    # First stage (straight to death) event simulations. While survival times through 
    # stage 1 are generated in continuous time, deaths in this stage are coded as left 
    # censored events assigned a time of 1 (see text S4 for detail). Alternatively, exact 
    # event times in this stage could also be used in which case deaths would be treated
    # as observed events (i.e. not left censorship events).
      temp1 <- simsurv(dist="exponential",x=data.frame(id=1),
            lambda = exp( log(lambda.v[1]) + data.df$Size[i]*beta_1))$eventtime
      # If death occurs in stage 1, record a left censorship event.
      if(temp1 <= a.v[2]) {data.df$Time[i] <- a.v[2]; data.df$Omega[i] <- 1; next}            
    # Second stage event simulations for subjects that survive beyond stage 1. First
    # generate the specimen's transition time out of stage 2.
      a3.temp <- a.v[2] + exp(log(tau2) + data.df$Size[i]*zeta)
      temp2 <- a.v[2] + simsurv(dist="exponential",x=data.frame(id=1),
            lambda = exp( log(lambda.v[2]) + data.df$Size[i]*beta_2))$eventtime
      if(temp2 <= a3.temp ) {data.df$Time[i] <- temp2; next}
    # Third stage event simulations for subjects that survive beyond stage 2  
      temp3 <- a3.temp + simsurv(dist="exponential",x=data.frame(id=1),
            lambda = exp( log(lambda.v[3]) + data.df$Size[i]*beta_3))$eventtime                    
      if(temp3 <= a.v[4]) {data.df$Time[i] <- temp3; next}
    # Record a terminal right censorship event for subjects that survive to 
    # end of the observation period 
      if(temp3 > a.v[4]){data.df$Time[i] <- a.v[4]; data.df$Delta[i] <- 1}
    }
```

Subsequently, specify survvial stage structure, priors, JAGS run
parameters, and package data for a 3-stage survival model with covariate
effects on stage-specific survival and on the time spent in stage 2
(i.e. the ‘acclimation’ period):

```
# User specifies-------------------------------------------------------------- 
  # Time interval cutpoints
    m <- 3 # Number of survival stages (subsequently, m+1 cutpoints)
    a1 <- 0 # Fix the first time interval cutpoint at 0
    a2 <- a.v[2] # Second cutpoint, defines exit of the first time stage
    # a3 The time spent in (and thus the exit time out of) the second 'acclimation' stage is estimated
    a4 <- a.v[4] # Fourth cutpoint, defines end of third stage
  # Covariate matrices
    X1 <- model.matrix(~-1+Size,data=data.df) # Covariate matrix for survival stage 1
    X2 <- model.matrix(~-1+Size,data=data.df) # Covariate matrix for stage 2
    X3 <- model.matrix(~-1+Size,data=data.df) # Covariate matrix for stage 3
    Z <- model.matrix(~-1+Size,data=data.df) # Covariate matrix for the estimated cutpoint (a3, i.e. 
                                             # exit from the second 'acclimation' stage)    
  # Prior specifications
    # Hazard rates
      # Baseline hazards: vague uniform priors for survival stages
        lambda_L.v <- c(0.0001, 0.0001, 0.0001) # Associated with upper limit on survival through a stage
        lambda_U.v <- c(2.0, 2.0, 2.0) # Associated with lower limit on survival through a stage      
      # Beta's for covariate effects on lambda in stage 1: vague uniform priors
        coeff_lambda1_L.v <- c(-0.2)
        coeff_lambda1_U.v <- c(0.2)
      # Beta's for covariate effects on lambda in stage 2: vague uniform priors
        coeff_lambda2_L.v <- c(-0.2)
        coeff_lambda2_U.v <- c(0.2)
      # Beta's for covariate effects on lambda in stage 3: vague uniform priors
        coeff_lambda3_L.v <- c(-0.2)
        coeff_lambda3_U.v <- c(0.2)
    # Time spent in the second 'acclimation' stage
      # Baseline: vague uniform prior
        tran_time_prior_L.v <- c(5) # Lower limit baseline time in stage 2
        tran_time_prior_U.v <- c(100) # Upper limit baseline time in stage 2
      # Coefficient for covariate on time in stage 2: vague uniform prior
        coeff_tran_L.v <- c(-0.2)
        coeff_tran_U.v <- c(0.2)          
  # JAGS model fit specifications     
    n_chains <- 3 # Number of chains
    n_adapt <- 1000 # Iterations for tuning JAGS adaptation phase
    n_update_iter <- 5000 # Burn in samples
    n_sample_iter <- 5000 # Posterior samples
    n_sample_thin <- 20 # Thin rate
  # Specify JAGS nodes to store  
    p.jags <- c(
    # Estimated parameters (initial values needed)
      "lambda","beta1","beta2","beta3","zeta","tran_time_base",
    # Derived parameters (no initial values needed)
      "deviance") # Hand-calculated deviance is included in model scripts for DIC-based model selection
  # Specify initial values for estimated parameters; add small deviations from true values
    i.jags <- function() {list(lambda = lambda.v * runif(n = 3, min = 0.9, max = 1.1),
        beta1 = beta_1+runif(1,-.005,.005),beta2 = beta_2+runif(1,-.005,.005), 
        beta3 = beta_3+runif(1,-.005,.005),tran_time_base = tau2+runif(1,-2,2), 
        zeta = zeta+runif(1,-.005,.005))}         
# End user specifies ----------------------------------------------------------

# Packaging for JAGS runs
  # Event time data
    times <- data.df$Time # Observed event time data, numeric vector
    times_2 <- data.df$Time_2 # Upper censor time only for interval censor events, 0 otherwise, numeric vector
    delta <- data.df$Delta # Observed censorship codes (1=right censorship event time, 0=otherwise), numeric vector  
    omega <- data.df$Omega # Observed censorship codes (1=left censorship event time, 0=otherwise), numeric vector  
    rho <- data.df$Rho # Observed censorship codes (1=interval censorship event time, 0=otherwise), numeric vector  
  # Create a list for JAGS
    d.jags <- list( 
      # Data and survival stage structure parameters
        times = times, times_2 = times_2, delta = delta, omega = omega, rho = rho,
        K = m, n = length(times), a1 = a1, a2 = a2, a4 = a4, X1 = X1, X2 = X2, X3 = X3, Z = Z, 
      # Priors and related parameter dimensions       
        lambda_L.v = lambda_L.v, lambda_U.v = lambda_U.v, 
        coeff_lambda1_L.v = coeff_lambda1_L.v, coeff_lambda1_U.v = coeff_lambda1_U.v,
        coeff_lambda2_L.v = coeff_lambda2_L.v, coeff_lambda2_U.v = coeff_lambda2_U.v,
        coeff_lambda3_L.v = coeff_lambda3_L.v, coeff_lambda3_U.v = coeff_lambda3_U.v,
        tran_time_prior_L.v = tran_time_prior_L.v,tran_time_prior_U.v = tran_time_prior_U.v, 
        coeff_tran_L.v = coeff_tran_L.v, coeff_tran_U.v = coeff_tran_U.v, 
        # Numbers of estimated coefficients
        Nbetas1 = ncol(X1), Nbetas2 = ncol(X2), Nbetas3 = ncol(X3), Nzeta = ncol(Z), 
      # Placeholder zeros and a constant 'C_pois' for the zeros likelihood 
      # specification trick
        zeros = rep(0, length(times)), C_pois = 100000
      ) # End d.jags
```

Finally, we fit the JAGS model and explore output.

```
# Fit the JAGS model (Model A, Text S5)
  mod <- jags.model(data = d.jags, 
        file = "ThreeStageSurvivalModelWithCovsStage2LengthEst.txt",
        inits = i.jags, n.chains = n_chains, n.adapt = n_adapt) # Compile the model
  update(mod, n_update_iter) # Burn in
  mcmc_samples <- coda.samples(mod, variable.names = p.jags, n.iter = n_sample_iter, thin = n_sample_thin) 

# Explore convergence, posterior summaries, and DIC
  gelman.diag(mcmc_samples) # R hat statistics
  summary(mcmc_samples)
  dev.v <- as.mcmc(do.call(rbind,mcmc_samples))[,"deviance"]
  mean(dev.v)+var(dev.v)/2 # DIC using 'pV' penalty term
```

### **Text S2: Multistage time-to-event cisco *(Coregonus artedi)* case study vignette**

Here we demonstrate data processing and model fitting for the top
DIC-supported model for the cisco case study. We present code to
implement a 3-stage survival model with subject **Age** or
**Length** effects on stage-specific survival rates and on
the transition time out of the second, i.e. “acclimation”, stage. The
first survival stage is cast as a unit time interval and represents a
“straight to death” zero inflation process. Model scripts are provided
in **Text S5-Model A**.

First, load libraries and read in the cisco case study.
**Length** data are mean-centered to avoid identifiability
problems with subject size effect coefficients, and subject
**Age** data are translated into dummy variables.

```
# setwd("Your working directory here") # Set the directory to the location for JAGS models and case study data.
library(rjags)
library(HDInterval)

# Get data  
  dat.df <- read.csv("CiscoData_SuppMat.csv", colClasses = c("integer", rep("factor",2), rep("numeric",6))) 
# Add columns of dummy variables for the 'Age' factor. Under proportional hazards models, 
# one level is wrapped into the baseline hazard rate.
  dat.df <- cbind(dat.df, model.matrix(~-1+ReleaseAge,data = dat.df)) 
# Mean-center length data to avoid hazard rate identifiability issues
  dat.df$ReleaseLength_mm_center <- dat.df$ReleaseLength_mm - mean(dat.df$ReleaseLength_mm)
```

Subsequently, specify survival stage structure, priors, JAGS run
parameters, and package data for the JAGS model. Here is the setup for a
3-stage model with categorical **Age** effects on survivals
and on the transition time out of the second ‘acclimation’ stage:

```
# User specifies-------------------------------------------------------------- 
  # Time interval cutpoints
    m <- 3 # Number of survival stages (subsequently, m+1 cutpoints)
    a1 <- 0 # Fix the first time interval cutpoint at 0
    a2 <- 1 # Second cutpoint, defines exit of the first stage, here a unit time 'straight to death' stage
    # a3 The time spent in (and thus the exit time out of) the second 'acclimation' stage is estimated
    a4 <- 999 # 4th and final cutpoint, set suitably large to exceed the maximum observed time
  # Covariate matrices (here in prep for model w/ subject length effects)
    X1 <- model.matrix(~-1+ReleaseAge1, data = dat.df)
    X2 <- model.matrix(~-1+ReleaseAge1, data = dat.df)
    X3 <- model.matrix(~-1+ReleaseAge1, data = dat.df)
    Z <- model.matrix(~-1+ReleaseAge1, data = dat.df)
  # Prior specifications
    # Hazard rates
      # Baseline hazards: vague uniform priors for survival stages of varying lengths
        lambda_L.v <- c(0.01, 0.001, 0.001)
        lambda_U.v <- c(5, 0.6, 0.6)    
      # Beta's for covariate effects on lambda in stage 1: vague uniform priors
        coeff_lambda1_L.v <- c(-5)
        coeff_lambda1_U.v <- c(5)  
      # Beta's for covariate effects on lambdas in stage 2: vague uniform priors
        coeff_lambda2_L.v <- c(-5)
        coeff_lambda2_U.v <- c(5)  
      # Beta's for covariate effects on lambda in stage 3: vague uniform priors
        coeff_lambda3_L.v <- c(-5)
        coeff_lambda3_U.v <- c(5)  
    # Time spent in the second 'acclimation' stage
      # Baseline: vague uniform prior
        tran_time_prior_L.v <- c(10)
        tran_time_prior_U.v <- c(70)
      # Coefficient for covariate on time in stage 2: vague uniform prior
        coeff_tran_L.v <- c(-3)
        coeff_tran_U.v <- c(3)    
  # JAGS model fit specifications     
    n_chains <- 3
    n_adapt <- 1000
    n_update_iter <- 5000
    n_sample_iter <- 5000  
    n_sample_thin <- 20
  # Specify JAGS nodes to store  
    p.jags <- c(
    # Estimated parameters (initial values needed)
      "lambda","beta1","beta2","beta3","zeta","tran_time_base",
    # Derived parameters (no initial values needed)
      "deviance")
  # Specify initial values for estimated parameters, best guesses here
    i.jags <- function() {list(lambda = c(0.8,0.04,0.01),
        beta1 = -1.8, beta2 = -1.4, beta3 = -0.5,
        zeta = 0.9,tran_time_base = 40)}   
# End user specifies ----------------------------------------------------------

# Packaging for JAGS runs
  # Event time data
    times <- dat.df$Time
    times_2 <- dat.df$Time_2
    delta <- dat.df$Delta  
    omega <- dat.df$Omega  
    rho <- dat.df$Rho  
  # Create a list for JAGS
    d.jags <- list( 
      # Data and survival stage structure parameters
        times = times, times_2 = times_2, delta = delta, omega = omega, rho = rho,
        K = m, n = length(times), a1 = 0, a2 = a2, a4 = a4, X1 = X1, X2 = X2, X3 = X3, Z = Z, 
      # Priors and related parameter dimensions       
        lambda_L.v = lambda_L.v, lambda_U.v = lambda_U.v, 
        coeff_lambda1_L.v = coeff_lambda1_L.v, coeff_lambda1_U.v = coeff_lambda1_U.v, 
        coeff_lambda2_L.v = coeff_lambda2_L.v, coeff_lambda2_U.v = coeff_lambda2_U.v, 
        coeff_lambda3_L.v = coeff_lambda3_L.v, coeff_lambda3_U.v = coeff_lambda3_U.v, 
        tran_time_prior_L.v = tran_time_prior_L.v, tran_time_prior_U.v = tran_time_prior_U.v, 
        coeff_tran_L.v = coeff_tran_L.v, coeff_tran_U.v = coeff_tran_U.v, 
        # Numbers of estimated coefficients
        Nbetas1 = ncol(X1), Nbetas2 = ncol(X2), Nbetas3 = ncol(X3), Nzeta = ncol(Z), 
      # Placeholder zeros and a constant 'C_pois' for the zeros likelihood 
      # specification trick
        zeros = rep(0, length(times)), C_pois = 100000
      ) # End d.jags
```

Finally, we fit the JAGS model and explore output:

```
# Fit the JAGS model (Model A, Text S5)
  mod <- jags.model(data = d.jags, 
        file = "ThreeStageSurvivalModelWithCovsStage2LengthEst.txt",
        inits = i.jags, n.chains = n_chains, n.adapt = n_adapt)
  update(mod, n_update_iter)
  mcmc_samples <- coda.samples(mod, variable.names = p.jags, n.iter = n_sample_iter, thin = n_sample_thin)

# Explore convergence, posterior summaries, DIC
  gelman.diag(mcmc_samples) # R hat statistics
  summary(mcmc_samples)
  dev.v <- as.mcmc(do.call(rbind,mcmc_samples))[,"deviance"]
  mean(dev.v)+var(dev.v)/2 # DIC using 'pV' penalty term

# Example derived parameter of interest with credibility intervals
  # Equivalent discrete annual survival associated with stage 3 instantaneous survival
    beta3 <- as.numeric(unlist(mcmc_samples[,"beta3"]))
    lambda3 <- as.numeric(unlist(mcmc_samples[,"lambda[3]"]))
    # Age 0
      median(exp( -1*(365*lambda3*exp(0*beta3))) )
      hdi(exp( -1*(365*lambda3*exp(0*beta3))),0.95)
    # Age 1
      median(exp( -1*(365*lambda3*exp(1*beta3))) )
      hdi(exp( -1*(365*lambda3*exp(1*beta3))),0.95)
  # Time spent in the second 'acclimation' stage
    zeta <- as.numeric(unlist(mcmc_samples[,"zeta"]))
    tran_time_base <- as.numeric(unlist(mcmc_samples[,"tran_time_base"]))
    # Age 0
        median(tran_time_base*exp(0*zeta))
        hdi(tran_time_base*exp(0*zeta),0.95) 
    # Age 1
        median(tran_time_base*exp(1*zeta))
          hdi(tran_time_base*exp(1*zeta),0.95)
```

For comparison, here is code to set up inputs for a 3-stage model
with subject **Length** effects (a continuous covariate) on
survivals and on the transition time out of the second “acclimation”
stage:

```
# User specifies-------------------------------------------------------------- 
  # Time interval cutpoints
    m <- 3 # Number of survival stages (subsequently, m+1 cutpoints)
    a1 <- 0 # Fix the first interval cutpoint at 0
    a2 <- 1 # Second cutpoint, defines exit of the first stage, here a unit time 'straight to death' stage
    # a3 The time spent in (and thus the exit time out of) the second 'acclimation' stage is estimated
    a4 <- 999 # Fourth and final cutpoint, set suitably large to exceed the maximum observed time
  # Covariate matrices (here in prep for model w/ subject length effects)
    X1 <- model.matrix(~-1+ReleaseLength_mm_center, data = dat.df)
    X2 <- model.matrix(~-1+ReleaseLength_mm_center, data = dat.df)
    X3 <- model.matrix(~-1+ReleaseLength_mm_center, data = dat.df)
    Z <- model.matrix(~-1+ReleaseLength_mm_center, data = dat.df)
  # Prior specifications
    # Hazard rates
      # Baseline hazards: vague uniform priors for survival stages of varying lengths
        lambda_L.v <- c(0.01, 0.001, 0.001)
        lambda_U.v <- c(5, 0.6, 0.6)    
      # # Beta's for covariate effects on lambdas: vague uniform priors
      # Beta's for covariate effects on lambda in stage 1: vague uniform priors
        coeff_lambda_L.v <- c(-0.25)
        coeff_lambda_U.v <- c(0.25)  
      # Beta's for covariate effects on lambda in stage 2: vague uniform priors
        coeff_lambda_L.v <- c(-0.25)
        coeff_lambda_U.v <- c(0.25)  
      # Beta's for covariate effects on lambda in stage 3: vague uniform priors
        coeff_lambda_L.v <- c(-0.25)
        coeff_lambda_U.v <- c(0.25)  
    # Time spent in the second 'acclimation' stage
      # Baseline: vague uniform prior
        tran_time_prior_L.v <- c(10)
        tran_time_prior_U.v <- c(70)
      # Coefficient for covariate on time in stage 2: required a somewhat less vague uniform prior
        coeff_tran_L.v <- c(-0.1) 
        coeff_tran_U.v <- c(0.1)  
  # JAGS model fit specifications (low sample iterations for demonstration purposes)     
    n_chains <- 3
    n_adapt <- 1000
    n_update_iter <- 5000
    n_sample_iter <- 5000  
    n_sample_thin <- 20
  # Specify JAGS nodes to store  
    p.jags <- c(
    # Estimated parameters (initial values needed)
      "lambda","beta1","beta2","beta3","zeta","tran_time_base",
    # Derived parameters (no initial values needed)
      "deviance")
  # Specify initial values for estimated parameters, best guesses here
    i.jags <- function() {list(lambda = c(0.8,0.04,0.01),
        beta1 = -1.95e-2, beta2 = -1.499e-2, beta3 = -1.625e-3,
        zeta = -4.1e-3,tran_time_base = 40)} 
# End user specifies ----------------------------------------------------------
```

### **Text S3: Multistage time-to-event wild turkey *(Meleagris gallopavo)* case study vignette**

Here we demonstrate data processing and model fitting for the top
DIC-supported model for the turkey case study. We present code to
implement a 2-stage survival model with subject release
**Weight** effects only on the first “capture impacts”
survival stage and where the length of the “capture impacts” stage is
also estimated. To implement this model, we will use the model script
for the most general 2-stage model with covariate effects on
stage-specific survivals and on the length of the first survival stage
(**Text S5-Model E**); however, we will specify dummy model
matrices and fix unutilized coefficients arbitrarily close to zero
through strong priors for model components that lack covariates. While
this trades off some computation time in fitting a larger model, it
avoids the necessity of writing additional scripts for nested
models.

First, load libraries and read in the turkey case study. Release
**Weight** data are mean-centered to avoid identifiability
problems with subject size effect coefficients.

```
# setwd("Your working directory here") # Set the directory to the location for JAGS models and case study data.
library(rjags)
library(HDInterval)

# Get data  
  dat.df <- read.csv("TurkeyData_SuppMat.csv",colClasses=c(rep("numeric",7)))   
# Mean-center length data to avoid hazard rate identifiability issues
  dat.df$ReleaseWeight_center <- dat.df$ReleaseWeight - mean(dat.df$ReleaseWeight)
```

Subsequently, specify survival stage structure, priors, JAGS run
parameters, and package data for the JAGS model:

```
# User specifies-------------------------------------------------------------- 
  # Time interval cutpoints
    m <- 2 # Number of survival stages (subsequently, m+1 cutpoints)
    a1 <- 0 # Fix the first interval cutpoint at 0
    # a2 The time spent in (and thus the exit time out of) the first 'capture impacts' stage is estimated
    a3 <- 999 # Third and final cutpoint, set suitably large to exceed the maximum observed time
  # Covariate matrices (here we include covariates on the hazard rate only in stage 1 and we 
  # create placeholder model matrices for stage 2 (X2) and the length of stage 1 (Z)).
    X1 <- model.matrix(~-1+ReleaseWeight_center,data=dat.df) # Release weight effects on stage 1 survival
    X2 <- model.matrix(~-1+X2,data=data.frame(X2=rep(0,nrow(dat.df)))) # Placeholder dummy model matrix 
    Z <- model.matrix(~-1+Z,data=data.frame(Z=rep(0,nrow(dat.df)))) # Placeholder dummy model matrix   
  # Prior specifications
    # Hazard rates
      # Baseline hazards: vague uniform priors for survival stages of varying lengths
        lambda_L.v <- c(0.00001, 0.00001)
        lambda_U.v <- c(0.5, 0.25)    
      # Beta's for covariate effects of release weight on lambda in stage 1: vague uniform priors. 
        coeff_lambda1_L.v <- c(-4)
        coeff_lambda1_U.v <- c(4)  
      # Beta's for covariate effects on lambda in stage 2: none modeled, use an informed uniform prior about zero
        coeff_lambda2_L.v <- c(-0.00001)
        coeff_lambda2_U.v <- c(0.00001)  
      # Time spent in the first 'capture myopathy' stage
      # Baseline: vague uniform prior
        tran_time_prior_L.v <- c(2)
        tran_time_prior_U.v <- c(40)
      # Coefficient for covariate on time in stage 1: none modeled, use an informed uniform prior about zero
        coeff_tran_L.v <- c(-0.00001) 
        coeff_tran_U.v <- c(0.00001)  
        
  # JAGS model fit parameters     
    n_chains <- 3
    n_adapt <- 1000
    n_update_iter <- 5000
    n_sample_iter <- 5000  
    n_sample_thin <- 20
  # Specify JAGS nodes to store  
    p.jags <- c(
    # Estimated parameters (initial values needed)
      "lambda","beta1","tran_time_base",
    # Derived parameters (no initial values needed)
      "deviance")
  # Specify initial values for estimated parameters, best guesses here
    i.jags <- function() {list(lambda = c(0.003, 0.0015),
        beta1 = 0, beta2 = 0, tran_time_base = 10, zeta = 0)}   
# End user specifies ----------------------------------------------------------

# Packaging for JAGS runs
  # Event time data
    times <- dat.df$Time
    times_2 <- dat.df$Time_2
    delta <- dat.df$Delta  
    omega <- dat.df$Omega  
    rho <- dat.df$Rho  
  # Create a list for JAGS
    d.jags <- list( 
      # Data and survival stage structure parameters
        times = times, times_2 = times_2, delta = delta, omega = omega, rho = rho,
        K = m, n = length(times), a1 = a1, a3 = a3, X1 = X1, X2 = X2, Z = Z,
      # Priors and related parameter dimensions       
        lambda_L.v = lambda_L.v, lambda_U.v = lambda_U.v, 
        coeff_lambda1_L.v = coeff_lambda1_L.v, coeff_lambda1_U.v = coeff_lambda1_U.v, 
        coeff_lambda2_L.v = coeff_lambda2_L.v, coeff_lambda2_U.v = coeff_lambda2_U.v, 
        tran_time_prior_L.v = tran_time_prior_L.v, tran_time_prior_U.v = tran_time_prior_U.v,
        coeff_tran_L.v = coeff_tran_L.v, coeff_tran_U.v = coeff_tran_U.v, 
      # Numbers of estimated coefficients
        Nbetas1 = ncol(X1), Nbetas2 = ncol(X2), Nzeta = ncol(Z), 
      # Placeholder zeros and a constant 'C_pois' for the zeros likelihood 
      # specification trick
        zeros = rep(0, length(times)), C_pois = 100000
      ) # End d.jags
```

Finally, we fit the JAGS model and explore output:

```
# Fit the JAGS model (Model E, Text S5)
  mod <- jags.model(data = d.jags, 
        file = "TwoStageSurvivalModelWithCovsStage1LengthEstimated.txt",
        inits = i.jags, n.chains = n_chains, n.adapt = n_adapt)
  update(mod, n_update_iter)
  mcmc_samples <- coda.samples(mod, variable.names = p.jags, n.iter = n_sample_iter, thin = n_sample_thin)

# Explore convergence, posterior summaries, DIC
  gelman.diag(mcmc_samples) # R hat statistics
  summary(mcmc_samples)
  dev.v <- as.mcmc(do.call(rbind,mcmc_samples))[,"deviance"]
  mean(dev.v)+var(dev.v)/2 # DIC using 'pV' penalty term

# Example derived parameter of interest
  # Expected survival through the stage 1 'capture effects' period
    tranbase <- as.numeric(unlist(mcmc_samples[,"tran_time_base"]))
    beta1 <- as.numeric(unlist(mcmc_samples[,"beta1"]))
    lam1 <- as.numeric(unlist(mcmc_samples[,"lambda[1]"]))
    # Credibility interval for the 50th percentile ReleaseWeight subject
      median(exp( -1*(tranbase * lam1*exp(quantile(dat.df$ReleaseWeight_center,0.5)*beta1))))
      hdi(exp( -1*(tranbase * lam1*exp(quantile(dat.df$ReleaseWeight_center,0.5)*beta1))),0.95)    
  # Ratio of stage 1 to stage 2 baseline hazard rate
    lam1 <- as.numeric(unlist(mcmc_samples[,"lambda[1]"]))
    lam2 <- as.numeric(unlist(mcmc_samples[,"lambda[2]"]))
    # Credibility interval
    median(lam1/lam2)
    hdi(lam1/lam2,0.95)
```

### **Text S4: Important points for fitting multistage time-to-event models**

1. The “zeros trick” in JAGS: JAGS lacks a probability distribution
   to describe the time-to-event likelihood outlined in equations 1-3 of
   the main text. However, we can use the zeros trick to specify any
   likelihood function. Functionally, to implement the zeros trick the user
   specifies a vector of zeros as pseudo-observations which are distributed
   Poisson with rate parameter equal to the customized negative log
   likelihood plus the addition of a large positive constant to ensure the
   Poisson mean remains positive.
2. Centering: Center continuous covariates to avoid identifiability
   issues when estimating coefficients.
3. Straight to death coding: When parameterizing a ‘straight to
   death’ interval for a multistage model, for example to represent a form
   of zero inflation for subjects that die immediately upon entry to a
   ‘trial’, care need be taken in the treatment of event times in this
   initial stage. If exact event times are available, then these data can
   be coded as directly observed events as usual. However, if instead the
   straight to death period is cast as a time window in which all event
   occurrences are treated as binary outcomes as having experienced the
   event during the period or not, then these records need be coded as left
   censorship events with a censorship time equal to the end of the
   straight to death period. Failure to treat events as left censorship
   events in such cases can induce bias in hazard rate estimates during
   this stage. This is because in time-to-event processes, subjects are
   exposed to a continuous time hazard and thus any events that occur over
   a time interval, even if short, are expected to follow a probabilistic
   process as opposed to all events occurring arbitrarily at the end of the
   specified interval.
4. Nested models: When exploring covariate effects on survivals or
   stage lengths for a given survival stage structure, the need to write
   separate scripts for nested models can be avoided by implementing the
   most general model form with covariate effects on all survivals and
   stage lengths and then subsequently ‘knocking out’ unutilized parameters
   by setting their priors arbitrarily close to zero (see **Text
   S3**).

### **Text S5: Multistage time-to-event model scripts**

Here we provide model scripts and basic code to package data to fit a
suite of multistage survival models. This code is designed to work with
a survival time model object as formatted in **Text S1**.
Prior specifications reflect vague distributions with respect to the
simulated data generating process in **Text S1**.

*Model A: 3-stage model with a
unit time interval stage 1 (“straight to death”) and where the time
spent in stage 2 is estimated. Covariate effects are included on
stage-specific survivals and on the time spent in stage
2.*

See example annotated code in Text S1 for packaging inputs to fit the
3-stage survival model with covariate effects on stage-specific
survivals and on the time spent in stage 2.

```
# Model script
cat("
model{
  #Priors
    #Baseline estimated time in stage 2
      tran_time_base ~ dunif(tran_time_prior_L.v[1],tran_time_prior_U.v[1]) 
    #Coefficients in linear predictor for time in stage 2
      for(p in 1:Nzeta){
        zeta[p]~dunif(coeff_tran_L.v[p],coeff_tran_U.v[p])
      }      
    #Baseline hazard rates
      for(k in 1:K){
        lambda[k]~dunif(lambda_L.v[k],lambda_U.v[k])
      }
    #Coefficients in linear predictors for baseline hazard rates  
      for(p in 1:Nbetas1){
        beta1[p]~dunif(coeff_lambda1_L.v[p],coeff_lambda1_U.v[p])
      }
      for(p in 1:Nbetas2){
        beta2[p]~dunif(coeff_lambda2_L.v[p],coeff_lambda2_U.v[p])
      }  
      for(p in 1:Nbetas3){
        beta3[p]~dunif(coeff_lambda3_L.v[p],coeff_lambda3_U.v[p])
      }
      
  #Assemble components for the likelihood
    for(i in 1:n){
      #Define time interval cutpoints
        #Linear predictor with covariate effects on length of stage 2
          elinpred_trantime[i] <- exp(inprod(zeta[],Z[i,])) 
        #Constraint to ensure the estimated transition time falls within a2 and a4
          tran_time[i] <- min(a4-1, a2 + tran_time_base*elinpred_trantime[i]) 
        #a1,a2,a4 passed to JAGS, here 'a3' is an estimated transition time  
          a_jags[i,1:(K+1)] <- c(a1,a2,tran_time[i],a4) 
      #Determine in which stage a given event time falls and calculate 
      #within stage cumulative times 
        #For these cases: observed event, left censor, right censor, and earliest 
        #of the pair of interval censor times
          for(k in 1:K) {
            #Logical tests
              test_gt_k[i,k] <- ifelse(times[i] > a_jags[i,k],1,0) #time>cutpoint k
              test_ltet_kplus1[i,k] <- ifelse(times[i] <= a_jags[i,k+1],1,0) #time<=k+1
              test_gt_kplus1[i,k] <- ifelse(times[i] > a_jags[i,k+1],1,0) #time>cutpoint k+1
            #Time falls within this stage? 1 = yes, 0 = no
              int_obs_m[i,k] <- test_gt_k[i,k]*test_ltet_kplus1[i,k]
            #Record time within stage.  time[i] w/in stage => cumulative time, else
            #time > end of this stage => total stage time, else record a 0.
              R[i,k] <- ifelse(int_obs_m[i,k]==1,
                    (times[i]-a_jags[i,k]),test_gt_kplus1[i,k]*(a_jags[i,k+1]-a_jags[i,k]))
          }
          #Track the stage number in which time[i] falls
            int_obs_v[i] <- inprod(int_obs_m[i,1:K] , 1:K) 
        #For these cases: latter of the pair of interval censor times
          for(k in 1:K) {
              test_gt_k_2[i,k] <- ifelse(times_2[i] > a_jags[i,k],1,0)
              test_ltet_kplus1_2[i,k] <- ifelse(times_2[i] <= a_jags[i,k+1],1,0)
              test_gt_kplus1_2[i,k] <- ifelse(times_2[i] > a_jags[i,k+1],1,0)
              int_obs_m_2[i,k] <- test_gt_k_2[i,k]*test_ltet_kplus1_2[i,k]
              R_2[i,k] <- ifelse(int_obs_m_2[i,k]==1,
                    (times_2[i]-a_jags[i,k]),test_gt_kplus1_2[i,k]*(a_jags[i,k+1]-a_jags[i,k]))
          }
      #Prepopulate stage-specific linear predictors for lambdas
        elinpred[i,1] <- exp(inprod(beta1[],X1[i,])) 
        elinpred[i,2] <- exp(inprod(beta2[],X2[i,]))
        elinpred[i,3] <- exp(inprod(beta3[],X3[i,]))
      #Populate pieces of the cumulative hazard by stage
        for(k in 1:K){
          #For observed event, left-, right-, and earlier of interval censor times
            haz_m[i,k] <- elinpred[i,k]*lambda[k]*R[i,k] 
          #Only for the latter of pair of censor times for an interval censor event
            haz_m_2[i,k] <- elinpred[i,k]*lambda[k]*R_2[i,k] 
        }
      #Cumulative hazard functions
        H[i] <- sum(haz_m[i,1:K])
        H_2[i] <- sum(haz_m_2[i,1:K])
    }
    
  #Likelihood
    for(i in 1:n){
      #Log hazard function: proportional hazards effects lam_k*exp(X_k'B_k)
        log_h[i] <- log(lambda[int_obs_v[i]]*elinpred[i,int_obs_v[i]]) 
      #Log survival function: S=exp(-H(t))
        log_S[i] <- -H[i] 
      #Log of (1-survival) function
        log_minus_S[i] <- log(1-exp(-H[i]))
      #Log of S_time1 (lower) minus log of S_time2 (upper) for an interval censor event 
      #ifelse() to avoid log(0) when an event is NOT an interval censor, i.e. time_2=0
        diff_S1_S2[i] <- ifelse(H_2[i]>0,exp(-H[i]) - exp(-H_2[i]), 1) 
        log_diff_S1_S2[i] <- log( diff_S1_S2[i] ) #Returns log(1)=0 if time_2=0
      #The log likelihood and zeros trick. Recall, indicators=1 if censorship occurs, 0 otherwise
        log_like[i] <- ( 
                #Observed event, f(t)=h(t)S(t), all censor indicators = 0
                  (1-delta[i])*(1-omega[i])*(1-rho[i])*(log_h[i]+log_S[i])
                #Right censor (delta=1), survival up to right censor time 
                  + delta[i]*(1-omega[i])*(1-rho[i])*log_S[i]          
                #Left censor (omega=1), cumulative mortality (1-S) to left censor time
                  + omega[i]*(1-delta[i])*(1-rho[i])*log_minus_S[i]      
                #Interval censor (rho=1), S to lower minus S to upper interval times 
                  + rho[i]*(1-delta[i])*(1-omega[i])*(log_diff_S1_S2[i]) 
                )  
        phi[i] <- C_pois - log_like[i] #Add a constant to ensure phi is positive
        zeros[i] ~ dpois(phi[i]) #Zeros vector passed to JAGS
    }
  
  #Derived par. for deviance, e.g. useful for pV=var(deviance[])/2) DIC complexity term  
    deviance <- -2*sum(log_like[1:n])  
} #end model
", file="ThreeStageSurvivalModelWithCovsStage2LengthEst.txt")
```

*Model B: 3-stage model with a
unit time interval stage 1 (“straight to death”) and where the time
spent in stage 2 is estimated, but without any covariate effects
included.*

```
# Model script
cat("
model{
  # Priors
    # Baseline estimated time in stage 2
      tran_time_base ~ dunif(tran_time_prior_L.v[1],tran_time_prior_U.v[1])       
    # Baseline hazard rates
      for(k in 1:K){
        lambda[k]~dunif(lambda_L.v[k],lambda_U.v[k])
      }
    
  # Assemble components for the likelihood
    for(i in 1:n){
      # Define time interval cutpoints
        # Constraint to ensure the estimated transition time falls within a2 and a4
          tran_time[i] <- min(a4-1, a2 + tran_time_base) 
        # a1,a2,a4 passed to JAGS, here 'a3' is an estimated transition time  
          a_jags[i,1:(K+1)] <- c(a1,a2,tran_time[i],a4) 
      # Determine in which stage a given event time falls and calculate 
      # within stage cumulative times 
        # For these cases: observed event, left censor, right censor, and earliest 
        # of the pair of interval censor times
          for(k in 1:K) {
            # Logical tests
              test_gt_k[i,k] <- ifelse(times[i] > a_jags[i,k],1,0) # time>cutpoint k
              test_ltet_kplus1[i,k] <- ifelse(times[i] <= a_jags[i,k+1],1,0) # time<=k+1
              test_gt_kplus1[i,k] <- ifelse(times[i] > a_jags[i,k+1],1,0) # time>cutpoint k+1
            # Time falls within this stage? 1 = yes, 0 = no
              int_obs_m[i,k] <- test_gt_k[i,k]*test_ltet_kplus1[i,k]
            # Record time within stage  time[i] w/in stage => cumulative time, else
            # time > end of this stage => total stage time, else record a 0.
              R[i,k] <- ifelse(int_obs_m[i,k]==1,
                    (times[i]-a_jags[i,k]),test_gt_kplus1[i,k]*(a_jags[i,k+1]-a_jags[i,k]))
          }
          # Track the stage number in which time[i] falls
            int_obs_v[i] <- inprod(int_obs_m[i,1:K] , 1:K) 
        # For these cases: latter of the pair of interval censor times
          for(k in 1:K) {
              test_gt_k_2[i,k] <- ifelse(times_2[i] > a_jags[i,k],1,0)
              test_ltet_kplus1_2[i,k] <- ifelse(times_2[i] <= a_jags[i,k+1],1,0)
              test_gt_kplus1_2[i,k] <- ifelse(times_2[i] > a_jags[i,k+1],1,0)
              int_obs_m_2[i,k] <- test_gt_k_2[i,k]*test_ltet_kplus1_2[i,k]
              R_2[i,k] <- ifelse(int_obs_m_2[i,k]==1,
                    (times_2[i]-a_jags[i,k]),test_gt_kplus1_2[i,k]*(a_jags[i,k+1]-a_jags[i,k]))
          }
      # Populate pieces of the cumulative hazard by stage
        for(k in 1:K){
          # For observed event, left-, right-, and earlier of interval censor times
            haz_m[i,k] <- lambda[k]*R[i,k] 
          # Only for the latter of pair of censor times for an interval censor event
            haz_m_2[i,k] <- lambda[k]*R_2[i,k] 
        }
      # Cumulative hazard functions
        H[i] <- sum(haz_m[i,1:K])
        H_2[i] <- sum(haz_m_2[i,1:K])
    }
    
  # Likelihood
    for(i in 1:n){
      # Log hazard function
        log_h[i] <- log(lambda[int_obs_v[i]]) 
      # Log survival function: S=exp(-H(t))
        log_S[i] <- -H[i] 
      # Log of (1-survival) function
        log_minus_S[i] <- log(1-exp(-H[i]))
      # Log of S_time1 (lower) minus log of S_time2 (upper) for an interval censor event 
      # ifelse() to avoid log(0) when an event is NOT an interval censor, i.e. time_2=0
        diff_S1_S2[i] <- ifelse(H_2[i]>0,exp(-H[i]) - exp(-H_2[i]), 1) 
        log_diff_S1_S2[i] <- log( diff_S1_S2[i] ) # Returns log(1)=0 if time_2=0
      # The log likelihood and zeros trick. Recall, indicators=1 if censorship occurs, 0 otherwise
        log_like[i] <- ( 
                # Observed event, f(t)=h(t)S(t), all censor indicators = 0
                  (1-delta[i])*(1-omega[i])*(1-rho[i])*(log_h[i]+log_S[i])
                # Right censor (delta=1), survival up to right censor time 
                  + delta[i]*(1-omega[i])*(1-rho[i])*log_S[i]          
                # Left censor (omega=1), cumulative mortality (1-S) to left censor time
                  + omega[i]*(1-delta[i])*(1-rho[i])*log_minus_S[i]      
                # Interval censor (rho=1), S to lower minus S to upper interval times 
                  + rho[i]*(1-delta[i])*(1-omega[i])*(log_diff_S1_S2[i]) 
                )  
        phi[i] <- C_pois - log_like[i] # Add a constant to ensure phi is positive
        zeros[i] ~ dpois(phi[i]) # Zeros vector passed to JAGS
    }
  
  # Derived par. for deviance, e.g. useful for pV=var(deviance[])/2) DIC complexity term  
    deviance <- -2*sum(log_like[1:n])  
} # end model
", file="ThreeStageSurvivalNoCovsStage2LengthEst.txt")
```

Code to package data and fit the model.

```
# User specifies stages, any fixed cutpoints, and priors ------------------------- 
  # Time interval cutpoints
    m <- 3 # Number of survival stages (subsequently, m+1 cutpoints)
    a1 <- 0 # Fix the first interval cutpoint at 0
    a2 <- 1 # Second cutpoint, defines exit of the first time stage
    # a3 The time spent in (and thus the exit time out of) the second 'acclimation' stage is estimated
    a4 <- 999 # Fourth and final cutpoint, set suitably large to exceed the maximum observed time
  # Prior specifications
    # Hazard rates
      # Baseline hazards: vague uniform priors
        lambda_L.v <- c(0.0001, 0.0001, 0.0001)
        lambda_U.v <- c(2.0, 2.0, 2.0)      
    # Time spent in the second 'acclimation' stage
      # Baseline: vague uniform prior
        tran_time_prior_L.v <- c(5)
        tran_time_prior_U.v <- c(100)      
  # JAGS model fit specifications (low sample iterations for demonstration purposes)     
    n_chains <- 3
    n_adapt <- 100
    n_update_iter <- 500
    n_sample_iter <- 500
    n_sample_thin <- 2
  # Specify JAGS nodes to store  
    p.jags <- c(
    # Estimated parameters (initial values needed)
      "lambda","tran_time_base",
    # Derived parameters (no initial values needed)
      "deviance")
  # Specify initial values for estimated parameters
    i.jags <- function() {list(lambda = c(0.8,0.01,0.001) * runif(n=3,min=0.9,max=1.1),
        tran_time_base = 50 + runif(1,-2,2))}         
# End user specifies ----------------------------------------------------------

# Packaging for JAGS runs
  # Event time data
    times <- data.df$Time
    times_2 <- data.df$Time_2
    delta <- data.df$Delta 
    omega <- data.df$Omega
    rho <- data.df$Rho
  # Create a list for JAGS
    d.jags <- list( 
      # Data and survival stage structure parameters
        times = times, times_2 = times_2, delta = delta, omega = omega, rho = rho,
        K = m, n = length(times), a1 = a1, a2 = a2, a4 = a4,
      # Priors and related parameter dimensions       
        lambda_L.v = lambda_L.v, lambda_U.v = lambda_U.v,  
        tran_time_prior_L.v = tran_time_prior_L.v,tran_time_prior_U.v = tran_time_prior_U.v,         
      # Placeholder zeros and a constant 'C_pois' for the zeros likelihood 
      # specification trick
        zeros = rep(0, length(times)), C_pois = 100000
      ) # End d.jags
      
# Fit the JAGS model
  mod <- jags.model(data = d.jags, 
        file = "ThreeStageSurvivalNoCovsStage2LengthEst.txt",
        inits = i.jags, n.chains = n_chains, n.adapt = n_adapt)
  update(mod, n_update_iter)
  mcmc_samples <- coda.samples(mod, variable.names = p.jags, n.iter = n_sample_iter, thin = n_sample_thin)

# Explore convergence, posterior summaries, and DIC
  gelman.diag(mcmc_samples) # R hat statistics
  summary(mcmc_samples)
  dev.v <- as.mcmc(do.call(rbind,mcmc_samples))[,"deviance"]
  mean(dev.v)+var(dev.v)/2 # DIC using 'pV' penalty term
```

*Model C: 2-stage model with a
unit time interval stage 1 (“straight to death”). Covariate effects are
included on stage-specific survivals.*

```
# Model script
cat("
model{
  # Priors
    # Baseline hazard rates
      for(k in 1:K){
        lambda[k]~dunif(lambda_L.v[k],lambda_U.v[k])
      }
    # Coefficients in linear predictors for baseline hazard rates  
      for(p in 1:Nbetas1){
        beta1[p]~dunif(coeff_lambda1_L.v[p],coeff_lambda1_U.v[p])
      }
      for(p in 1:Nbetas2){
        beta2[p]~dunif(coeff_lambda2_L.v[p],coeff_lambda2_U.v[p])
      }  
      
  # Assemble components for the likelihood
    for(i in 1:n){
      # Define time interval cutpoints, here fixed cutpoints are passed to JAGS
        a_jags[i,1:(K+1)] <- c(a1,a2,a3) 
      # Determine in which stage a given event time falls and calculate 
      # within stage cumulative times 
        # For these cases: observed event, left censor, right censor, and earliest 
        # of the pair of interval censor times
          for(k in 1:K) {
            # Logical tests
              test_gt_k[i,k] <- ifelse(times[i] > a_jags[i,k],1,0) # time>cutpoint k
              test_ltet_kplus1[i,k] <- ifelse(times[i] <= a_jags[i,k+1],1,0) # time<=k+1
              test_gt_kplus1[i,k] <- ifelse(times[i] > a_jags[i,k+1],1,0) # time>cutpoint k+1
            # Time falls within this stage? 1 = yes, 0 = no
              int_obs_m[i,k] <- test_gt_k[i,k]*test_ltet_kplus1[i,k]
            # Record time within stage.  time[i] w/in stage => cumulative time, else
            # time > end of this stage => total stage time, else record a 0.
              R[i,k] <- ifelse(int_obs_m[i,k]==1,
                    (times[i]-a_jags[i,k]),test_gt_kplus1[i,k]*(a_jags[i,k+1]-a_jags[i,k]))
          }
          # Track the stage number in which time[i] falls
            int_obs_v[i] <- inprod(int_obs_m[i,1:K] , 1:K) 
        # For these cases: latter of the pair of interval censor times
          for(k in 1:K) {
              test_gt_k_2[i,k] <- ifelse(times_2[i] > a_jags[i,k],1,0)
              test_ltet_kplus1_2[i,k] <- ifelse(times_2[i] <= a_jags[i,k+1],1,0)
              test_gt_kplus1_2[i,k] <- ifelse(times_2[i] > a_jags[i,k+1],1,0)
              int_obs_m_2[i,k] <- test_gt_k_2[i,k]*test_ltet_kplus1_2[i,k]
              R_2[i,k] <- ifelse(int_obs_m_2[i,k]==1,
                    (times_2[i]-a_jags[i,k]),test_gt_kplus1_2[i,k]*(a_jags[i,k+1]-a_jags[i,k]))
          }
      # Prepopulate stage-specific linear predictors for lambdas
        elinpred[i,1] <- exp(inprod(beta1[],X1[i,])) 
        elinpred[i,2] <- exp(inprod(beta2[],X2[i,]))
      # Populate pieces of the cumulative hazard by stage
        for(k in 1:K){
          # For observed event, left-, right-, and earlier of interval censor times
            haz_m[i,k] <- elinpred[i,k]*lambda[k]*R[i,k] 
          # Only for the latter of pair of censor times for an interval censor event
            haz_m_2[i,k] <- elinpred[i,k]*lambda[k]*R_2[i,k] 
        }
      # Cumulative hazard functions
        H[i] <- sum(haz_m[i,1:K])
        H_2[i] <- sum(haz_m_2[i,1:K])
    }
    
  # Likelihood
    for(i in 1:n){
      # Log hazard function: proportional hazards effects lam_k*exp(X_k'B_k)
        log_h[i] <- log(lambda[int_obs_v[i]]*elinpred[i,int_obs_v[i]]) 
      # Log survival function: S=exp(-H(t))
        log_S[i] <- -H[i] 
      # Log of (1-survival) function
        log_minus_S[i] <- log(1-exp(-H[i]))
      # Log of S_time1 (lower) minus log of S_time2 (upper) for an interval censor event 
      # ifelse() to avoid log(0) when an event is NOT an interval censor, i.e. time_2=0
        diff_S1_S2[i] <- ifelse(H_2[i]>0,exp(-H[i]) - exp(-H_2[i]), 1) 
        log_diff_S1_S2[i] <- log( diff_S1_S2[i] ) # Returns log(1)=0 if time_2=0
      # The log likelihood and zeros trick. Recall, indicators=1 if censorship occurs, 0 otherwise
        log_like[i] <- ( 
                # Observed event, f(t)=h(t)S(t), all censor indicators = 0
                  (1-delta[i])*(1-omega[i])*(1-rho[i])*(log_h[i]+log_S[i])
                # Right censor (delta=1), survival up to right censor time 
                  + delta[i]*(1-omega[i])*(1-rho[i])*log_S[i]          
                # Left censor (omega=1), cumulative mortality (1-S) to left censor time
                  + omega[i]*(1-delta[i])*(1-rho[i])*log_minus_S[i]      
                # Interval censor (rho=1), S to lower minus S to upper interval times 
                  + rho[i]*(1-delta[i])*(1-omega[i])*(log_diff_S1_S2[i]) 
                )  
        phi[i] <- C_pois - log_like[i] # Add a constant to ensure phi is positive
        zeros[i] ~ dpois(phi[i]) # Zeros vector passed to JAGS
    }
  
  # Derived par. for deviance, e.g. useful for pV=var(deviance[])/2) DIC complexity term  
    deviance <- -2*sum(log_like[1:n])  
} # end model
", file="TwoStageSurvivalWithCovsUnitInterval1.txt")
```

Code to package data and fit the model.

```
# User specifies stages, any fixed cutpoints, and priors -------------------------  
  # Time interval cutpoints
    m <- 2 # Number of survival stages (subsequently, m+1 cutpoints)
    a1 <- 0 # Fix the first interval cutpoint at 0
    a2 <- 1 # Second cutpoint, defines exit of the first time stage, here a unit time 'straight to death' stage
    a3 <- 999 # Third and final cutpoint, set suitably large to exceed the maximum observed time
  # Covariate matrices
    X1 <- model.matrix(~-1+Size,data=data.df)
    X2 <- model.matrix(~-1+Size,data=data.df)
  # Prior specifications
    # Hazard rates
      # Baseline hazards: vague uniform priors
        lambda_L.v <- c(0.0001, 0.0001)
        lambda_U.v <- c(2.0, 2.0)      
      # Beta's for covariate effects on lambda in stage 1: vague uniform priors
        coeff_lambda1_L.v <- c(-0.25)
        coeff_lambda1_U.v <- c(0.25)        
      # Beta's for covariate effects on lambda in stage 2: vague uniform priors
        coeff_lambda2_L.v <- c(-0.25)
        coeff_lambda2_U.v <- c(0.25)   
  # JAGS model fit specifications (low sample iterations for demonstration purposes)        
    n_chains <- 3
    n_adapt <- 100
    n_update_iter <- 500
    n_sample_iter <- 500
    n_sample_thin <- 2
  # Specify JAGS nodes to store  
    p.jags <- c(
    # Estimated parameters (initial values needed)
      "lambda","beta1","beta2",
    # Derived parameters (no initial values needed)
      "deviance")
  # Specify initial values for estimated parameters
    i.jags <- function() {list(lambda = c(0.8,0.01) * runif(n=2,min=0.9,max=1.1),
        beta1 = 0 + runif(1,-0.005,0.005), beta2 = 0 + runif(1,-0.005,0.005) 
        )}         
# End user specifies ----------------------------------------------------------

# Packaging for JAGS runs
  # Event time data
    times <- data.df$Time
    times_2 <- data.df$Time_2
    delta <- data.df$Delta
    omega <- data.df$Omega  
    rho <- data.df$Rho
  # Create a list for JAGS
    d.jags <- list( 
      # Data and survival stage structure parameters
        times = times, times_2 = times_2, delta = delta, omega = omega, rho = rho,
        K = m, n = length(times), a1 = a1, a2 = a2, a3 = a3, X1 = X1, X2 = X2, 
      # Priors and related parameter dimensions       
        lambda_L.v = lambda_L.v, lambda_U.v = lambda_U.v, 
        coeff_lambda1_L.v = coeff_lambda1_L.v, coeff_lambda1_U.v = coeff_lambda1_U.v,
        coeff_lambda2_L.v = coeff_lambda2_L.v, coeff_lambda2_U.v = coeff_lambda2_U.v,
      # Numbers of estimated coefficients
        Nbetas1 = ncol(X1), Nbetas2 = ncol(X2),
      # Placeholder zeros and a constant 'C_pois' for the zeros likelihood 
      # specification trick
        zeros = rep(0, length(times)), C_pois = 100000
      ) # End d.jags
      
# Fit the JAGS model
  mod <- jags.model(data = d.jags, 
        file = "TwoStageSurvivalWithCovsUnitInterval1.txt",
        inits = i.jags, n.chains = n_chains, n.adapt = n_adapt)
  update(mod, n_update_iter)
  mcmc_samples <- coda.samples(mod, variable.names = p.jags, n.iter = n_sample_iter, thin = n_sample_thin)

# Explore convergence, posterior summaries, and DIC
  gelman.diag(mcmc_samples) # R hat statistics
  summary(mcmc_samples)
  dev.v <- as.mcmc(do.call(rbind,mcmc_samples))[,"deviance"]
  mean(dev.v)+var(dev.v)/2 # DIC using 'pV' penalty term
```

*Model D: 2-stage model with a
unit time interval stage 1 (“straight to death”), but without any
covariate effects included.*

```
# Model script
cat("
model{
  # Priors
    # Baseline hazard rates
      for(k in 1:K){
        lambda[k]~dunif(lambda_L.v[k],lambda_U.v[k])
      }
      
  # Assemble components for the likelihood
    for(i in 1:n){
      # Define time interval cutpoints, here fixed cutpoints are passed to JAGS
        a_jags[i,1:(K+1)] <- c(a1,a2,a3) 
      # Determine in which stage a given event time falls and calculate 
      # within stage cumulative times 
        # For these cases: observed event, left censor, right censor, and earliest 
        # of the pair of interval censor times
          for(k in 1:K) {
            # Logical tests
              test_gt_k[i,k] <- ifelse(times[i] > a_jags[i,k],1,0) # time>cutpoint k
              test_ltet_kplus1[i,k] <- ifelse(times[i] <= a_jags[i,k+1],1,0) # time<=k+1
              test_gt_kplus1[i,k] <- ifelse(times[i] > a_jags[i,k+1],1,0) # time>cutpoint k+1
            # Time within this stage? 1 = yes, 0 = no
              int_obs_m[i,k] <- test_gt_k[i,k]*test_ltet_kplus1[i,k]
            # Record time within stage.  time[i] w/in stage => cumulative time, else
            # time > end of this stage => total stage time, else record a 0.
              R[i,k] <- ifelse(int_obs_m[i,k]==1,
                    (times[i]-a_jags[i,k]),test_gt_kplus1[i,k]*(a_jags[i,k+1]-a_jags[i,k]))
          }
          # Track the stage number in which time[i] falls
            int_obs_v[i] <- inprod(int_obs_m[i,1:K] , 1:K) 
        # For these cases: latter of the pair of interval censor times
          for(k in 1:K) {
              test_gt_k_2[i,k] <- ifelse(times_2[i] > a_jags[i,k],1,0)
              test_ltet_kplus1_2[i,k] <- ifelse(times_2[i] <= a_jags[i,k+1],1,0)
              test_gt_kplus1_2[i,k] <- ifelse(times_2[i] > a_jags[i,k+1],1,0)
              int_obs_m_2[i,k] <- test_gt_k_2[i,k]*test_ltet_kplus1_2[i,k]
              R_2[i,k] <- ifelse(int_obs_m_2[i,k]==1,
                    (times_2[i]-a_jags[i,k]),test_gt_kplus1_2[i,k]*(a_jags[i,k+1]-a_jags[i,k]))
          }
      # Populate pieces of the cumulative hazard by stage
        for(k in 1:K){
          # For observed event, left-, right-, and earlier of interval censor times
            haz_m[i,k] <- lambda[k]*R[i,k] 
          # Only for the latter of pair of censor times for an interval censor event
            haz_m_2[i,k] <- lambda[k]*R_2[i,k] 
        }
      # Cumulative hazard functions
        H[i] <- sum(haz_m[i,1:K])
        H_2[i] <- sum(haz_m_2[i,1:K])
    }
    
  # Likelihood
    for(i in 1:n){
      # Log hazard function
        log_h[i] <- log(lambda[int_obs_v[i]]) 
      # Log survival function: S=exp(-H(t))
        log_S[i] <- -H[i] 
      # Log of (1-survival) function
        log_minus_S[i] <- log(1-exp(-H[i]))
      # Log of S_time1 (lower) minus log of S_time2 (upper) for an interval censor event 
      # ifelse() to avoid log(0) when an event is NOT an interval censor, i.e. time_2=0
        diff_S1_S2[i] <- ifelse(H_2[i]>0,exp(-H[i]) - exp(-H_2[i]), 1) 
        log_diff_S1_S2[i] <- log( diff_S1_S2[i] ) # Returns log(1)=0 if time_2=0
      # The log likelihood and zeros trick. Recall, indicators=1 if censorship occurs, 0 otherwise
        log_like[i] <- ( 
                # Observed event, f(t)=h(t)S(t), all censor indicators = 0
                  (1-delta[i])*(1-omega[i])*(1-rho[i])*(log_h[i]+log_S[i])
                # Right censor (delta=1), survival up to right censor time 
                  + delta[i]*(1-omega[i])*(1-rho[i])*log_S[i]          
                # Left censor (omega=1), cumulative mortality (1-S) to left censor time
                  + omega[i]*(1-delta[i])*(1-rho[i])*log_minus_S[i]      
                # Interval censor (rho=1), S to lower minus S to upper interval times 
                  + rho[i]*(1-delta[i])*(1-omega[i])*(log_diff_S1_S2[i]) 
                )  
        phi[i] <- C_pois - log_like[i] # Add a constant to ensure phi is positive
        zeros[i] ~ dpois(phi[i]) # Zeros vector passed to JAGS
    }
  
  # Derived par. for deviance, e.g. useful for pV=var(deviance[])/2) DIC complexity term  
    deviance <- -2*sum(log_like[1:n])  
} # end model
", file="TwoStageSurvivalModelNoCovsUnitInterval1.txt")
```

Code to package data and fit the model.

```
# User specifies stages, any fixed cutpoints, and priors ------------------------- 
  # Time interval cutpoints
    m <- 2 # Number of survival stages (subsequently, m+1 cutpoints)
    a1 <- 0 # Fix the first interval cutpoint at 0
    a2 <- 1 # Second cutpoint, defines exit of the first time stage, here a unit time 'straight to death' stage
    a3 <- 999 # Third and final cutpoint, set suitably large to exceed the maximum observed time
  # Prior specifications
    # Hazard rates
      # Baseline hazards: vague uniform priors
        lambda_L.v <- c(0.0001, 0.0001)
        lambda_U.v <- c(2.0, 2.0)          
  # JAGS model fit specifications (low sample iterations for demonstration purposes)     
    n_chains <- 3
    n_adapt <- 100
    n_update_iter <- 500
    n_sample_iter <- 500
    n_sample_thin <- 2
  # Specify JAGS nodes to store  
    p.jags <- c(
    # Estimated parameters (initial values needed)
      "lambda",
    # Derived parameters (no initial values needed)
      "deviance")
  # Specify initial values for estimated parameters
    i.jags <- function() {list(lambda = c(0.8,0.01) * runif(n=2,min=0.9,max=1.1)
        )}         
# End user specifies ----------------------------------------------------------

# Packaging for JAGS runs
  # Event time data
    times <- data.df$Time
    times_2 <- data.df$Time_2
    delta <- data.df$Delta
    omega <- data.df$Omega  
    rho <- data.df$Rho  
  # Create a list for JAGS
    d.jags <- list( 
      # Data and survival survival structure parameters
        times = times, times_2 = times_2, delta = delta, omega = omega, rho = rho,
        K = m, n = length(times), a1 = a1, a2 = a2, a3 = a3, 
      # Priors and related parameter dimensions       
        lambda_L.v = lambda_L.v, lambda_U.v = lambda_U.v, 
      # Placeholder zeros and a constant 'C_pois' for the zeros likelihood 
      # specification trick
        zeros = rep(0, length(times)), C_pois = 100000
      ) # End d.jags
      
# Fit the JAGS model
  mod <- jags.model(data = d.jags, 
        file = "TwoStageSurvivalModelNoCovsUnitInterval1.txt",
        inits = i.jags, n.chains = n_chains, n.adapt = n_adapt)
  update(mod, n_update_iter)
  mcmc_samples <- coda.samples(mod, variable.names = p.jags, n.iter = n_sample_iter, thin = n_sample_thin)

# Explore convergence, posterior summaries, and DIC
  gelman.diag(mcmc_samples) # R hat statistics
  summary(mcmc_samples)
  dev.v <- as.mcmc(do.call(rbind,mcmc_samples))[,"deviance"]
  mean(dev.v)+var(dev.v)/2 # DIC using 'pV' penalty term
```

*Model E: 2-stage model where the
time spent in stage 1 is estimated. Covariate effects are included on
stage-specific survivals and on the time spent in stage
1.*

```
# Model script
cat("
model{
  # Priors
    # Baseline estimated time in stage 1
      tran_time_base ~ dunif(tran_time_prior_L.v[1],tran_time_prior_U.v[1]) 
    # Coefficients in linear predictor for time in stage 1
      for(p in 1:Nzeta){
        zeta[p]~dunif(coeff_tran_L.v[p],coeff_tran_U.v[p])
      }      
    # Baseline hazard rates
      for(k in 1:K){
        lambda[k]~dunif(lambda_L.v[k],lambda_U.v[k])
      }
    # Coefficients in linear predictors for baseline hazard rates  
      for(p in 1:Nbetas1){
        beta1[p]~dunif(coeff_lambda1_L.v[p],coeff_lambda1_U.v[p])
      }
      for(p in 1:Nbetas2){
        beta2[p]~dunif(coeff_lambda2_L.v[p],coeff_lambda2_U.v[p])
      }  
      
  # Assemble components for the likelihood
    for(i in 1:n){
      # Define time interval cutpoints
        # Linear predictor with covariate effects on stage 1 length
          elinpred_trantime[i] <- exp(inprod(zeta[],Z[i,])) 
        # Constraint to ensure the estimated transition time falls within a2 and a3
          tran_time[i] <- min(a3-1, a1 + tran_time_base*elinpred_trantime[i]) 
        # a1,a3 passed to JAGS, here 'a2' is an estimated transition time  
          a_jags[i,1:(K+1)] <- c(a1,tran_time[i],a3) 
      # Determine in which stage a given event time falls and calculate 
      # within stage cumulative times 
        # For these cases: observed event, left censor, right censor, and earliest 
        # of the pair of interval censor times
          for(k in 1:K) {
            # Logical tests
              test_gt_k[i,k] <- ifelse(times[i] > a_jags[i,k],1,0) # time>cutpoint k
              test_ltet_kplus1[i,k] <- ifelse(times[i] <= a_jags[i,k+1],1,0) # time<=k+1
              test_gt_kplus1[i,k] <- ifelse(times[i] > a_jags[i,k+1],1,0) # time>cutpoint k+1
            # Time within this stage? 1 = yes, 0 = no
              int_obs_m[i,k] <- test_gt_k[i,k]*test_ltet_kplus1[i,k]
            # Record time within stage.  time[i] w/in stage => cumulative time, else
            # time > end of this stage => total stage time, else record a 0.
              R[i,k] <- ifelse(int_obs_m[i,k]==1,
                    (times[i]-a_jags[i,k]),test_gt_kplus1[i,k]*(a_jags[i,k+1]-a_jags[i,k]))
          }
          # Track the stage number in which time[i] falls
            int_obs_v[i] <- inprod(int_obs_m[i,1:K] , 1:K) 
        # For these cases: latter of the pair of interval censor times
          for(k in 1:K) {
              test_gt_k_2[i,k] <- ifelse(times_2[i] > a_jags[i,k],1,0)
              test_ltet_kplus1_2[i,k] <- ifelse(times_2[i] <= a_jags[i,k+1],1,0)
              test_gt_kplus1_2[i,k] <- ifelse(times_2[i] > a_jags[i,k+1],1,0)
              int_obs_m_2[i,k] <- test_gt_k_2[i,k]*test_ltet_kplus1_2[i,k]
              R_2[i,k] <- ifelse(int_obs_m_2[i,k]==1,
                    (times_2[i]-a_jags[i,k]),test_gt_kplus1_2[i,k]*(a_jags[i,k+1]-a_jags[i,k]))
          }
      # Prepopulate stage-specific linear predictors for lambdas
        elinpred[i,1] <- exp(inprod(beta1[],X1[i,])) 
        elinpred[i,2] <- exp(inprod(beta2[],X2[i,]))
      # Populate pieces of the cumulative hazard by stage
        for(k in 1:K){
          # For observed event, left-, right-, and earlier of interval censor times
            haz_m[i,k] <- elinpred[i,k]*lambda[k]*R[i,k] 
          # Only for the latter of pair of censor times for an interval censor event
            haz_m_2[i,k] <- elinpred[i,k]*lambda[k]*R_2[i,k] 
        }
      # Cumulative hazard functions
        H[i] <- sum(haz_m[i,1:K])
        H_2[i] <- sum(haz_m_2[i,1:K])
    }
    
  # Likelihood
    for(i in 1:n){
      # Log hazard function: proportional hazards effects lam_k*exp(X_k'B_k)
        log_h[i] <- log(lambda[int_obs_v[i]]*elinpred[i,int_obs_v[i]]) 
      # Log survival function: S=exp(-H(t))
        log_S[i] <- -H[i] 
      # Log of (1-survival) function
        log_minus_S[i] <- log(1-exp(-H[i]))
      # Log of S_time1 (lower) minus log of S_time2 (upper) for an interval censor event 
      # ifelse() to avoid log(0) when an event is NOT an interval censor, i.e. time_2=0
        diff_S1_S2[i] <- ifelse(H_2[i]>0,exp(-H[i]) - exp(-H_2[i]), 1) 
        log_diff_S1_S2[i] <- log( diff_S1_S2[i] ) # Returns log(1)=0 if time_2=0
      # The log likelihood and zeros trick. Recall, indicators=1 if censorship occurs, 0 otherwise
        log_like[i] <- ( 
                # Observed event, f(t)=h(t)S(t), all censor indicators = 0
                  (1-delta[i])*(1-omega[i])*(1-rho[i])*(log_h[i]+log_S[i])
                # Right censor (delta=1), survival up to right censor time 
                  + delta[i]*(1-omega[i])*(1-rho[i])*log_S[i]          
                # Left censor (omega=1), cumulative mortality (1-S) to left censor time
                  + omega[i]*(1-delta[i])*(1-rho[i])*log_minus_S[i]      
                # Interval censor (rho=1), S to lower minus S to upper interval times 
                  + rho[i]*(1-delta[i])*(1-omega[i])*(log_diff_S1_S2[i]) 
                )  
        phi[i] <- C_pois - log_like[i] # Add a constant to ensure phi is positive
        zeros[i] ~ dpois(phi[i]) # Zeros vector passed to JAGS
    }
  
  # Derived par. for deviance, e.g. useful for pV=var(deviance[])/2) DIC complexity term  
    deviance <- -2*sum(log_like[1:n])  
} # end model
", file="TwoStageSurvivalModelWithCovsStage1LengthEstimated.txt")
```

Code to package data and fit the model.

```
# User specifies stages, any fixed cutpoints, and priors ------------------------- 
  # Time interval cutpoints
    m <- 2 # Number of survival stages (subsequently, m+1 cutpoints)
    a1 <- 0 # Fix the first interval cutpoint at 0
    # a2 The time spent in (and thus the exit time out of) the first stage is estimated
    a3 <- 999 # Third and final cutpoint, set suitably large to exceed the maximum observed time 
  # Covariate matrices
    X1 <- model.matrix(~-1+Size,data=data.df)
    X2 <- model.matrix(~-1+Size,data=data.df)
    Z <- model.matrix(~-1+Size,data=data.df)
  # Prior specifications
    # Hazard rates
      # Baseline hazards: vague uniform priors
        lambda_L.v <- c(0.0001, 0.0001)
        lambda_U.v <- c(2.0, 2.0)      
      # Beta's for covariate effects on lambdas: vague uniform priors
        coeff_lambda1_L.v <- c(-0.25)
        coeff_lambda1_U.v <- c(0.25)  
      # Beta's for covariate effects on lambdas: vague uniform priors
        coeff_lambda2_L.v <- c(-0.25)
        coeff_lambda2_U.v <- c(0.25)  
      # Time spent in the first survival stage
      # Baseline: vague uniform prior
        tran_time_prior_L.v <- c(2)
        tran_time_prior_U.v <- c(100)
      # Coefficient for covariate on time in stage 1: vague uniform prior
        coeff_tran_L.v <- c(-0.2)
        coeff_tran_U.v <- c(0.2)          
  # JAGS model fit specifications (low sample iterations for demonstration purposes)    
    n_chains <- 3
    n_adapt <- 100
    n_update_iter <- 500
    n_sample_iter <- 500
    n_sample_thin <- 2
  # Specify JAGS nodes to store  
    p.jags <- c(
    # Estimated parameters (initial values needed)
      "lambda","beta1","beta2","zeta","tran_time_base",
    # Derived parameters (no initial values needed)
      "deviance")
  # Specify initial values for estimated parameters
    i.jags <- function() {list(lambda = c(0.8,0.01) * runif(n=2,min=0.9,max=1.1),
        beta1 = 0 + runif(1,-0.005,0.005),beta2 = 0 + runif(1,-0.005,0.005),
        tran_time_base = 10 + runif(1,-2,2), zeta = 0 + runif(1,-0.005,0.005)
        )}         
# End user specifies ----------------------------------------------------------

# Packaging for JAGS runs
  # Event time data
    times <- data.df$Time
    times_2 <- data.df$Time_2
    delta <- data.df$Delta
    omega <- data.df$Omega  
    rho <- data.df$Rho
  # Create a list for JAGS
    d.jags <- list( 
      # Data and survival stage structure parameters
        times = times, times_2 = times_2, delta = delta, omega = omega, rho = rho,
        K = m, n = length(times), a1 = a1, a3 = a3, X1 = X1, X2 = X2, Z = Z, 
      # Priors and related parameter dimensions       
        lambda_L.v = lambda_L.v, lambda_U.v = lambda_U.v, 
        coeff_lambda1_L.v = coeff_lambda1_L.v, coeff_lambda1_U.v = coeff_lambda1_U.v, 
        coeff_lambda2_L.v = coeff_lambda2_L.v, coeff_lambda2_U.v = coeff_lambda2_U.v, 
        tran_time_prior_L.v = tran_time_prior_L.v, tran_time_prior_U.v = tran_time_prior_U.v, 
        coeff_tran_L.v = coeff_tran_L.v, coeff_tran_U.v = coeff_tran_U.v, 
        # Numbers of estimated coefficients
        Nbetas1 = ncol(X1), Nbetas2 = ncol(X2), Nzeta = ncol(Z), 
      # Placeholder zeros and a constant 'C_pois' for the zeros likelihood 
      # specification trick
        zeros = rep(0, length(times)), C_pois = 100000
      ) # End d.jags
      
# Fit the JAGS model
  mod <- jags.model(data = d.jags, 
        file = "TwoStageSurvivalModelWithCovsStage1LengthEstimated.txt",
        inits = i.jags, n.chains = n_chains, n.adapt = n_adapt)
  update(mod, n_update_iter)
  mcmc_samples <- coda.samples(mod, variable.names = p.jags, n.iter = n_sample_iter, thin = n_sample_thin)

# Explore convergence, posterior summaries, and DIC
  gelman.diag(mcmc_samples) # R hat statistics
  summary(mcmc_samples)
  dev.v <- as.mcmc(do.call(rbind,mcmc_samples))[,"deviance"]
  mean(dev.v)+var(dev.v)/2 # DIC using 'pV' penalty term
```

*Model F: 2-stage model where the
time spent in stage 1 is estimated, but without any covariate effects
included.*

```
# Model script
cat("
model{
  # Priors
    # Baseline estimated time in stage 1
      tran_time_base ~ dunif(tran_time_prior_L.v[1],tran_time_prior_U.v[1])     
    # Baseline hazard rates
      for(k in 1:K){
        lambda[k]~dunif(lambda_L.v[k],lambda_U.v[k])
      }
      
  # Assemble components for the likelihood
    for(i in 1:n){
      # Define time interval cutpoints
        # Constraint to ensure the estimated transition time falls within a2 and a3
          tran_time[i] <- min(a3-1, a1 + tran_time_base) 
        # a1,a3 passed to JAGS, here 'a2' is an estimated transition time  
          a_jags[i,1:(K+1)] <- c(a1,tran_time[i],a3) 
      # Determine in which stage a given event time falls and calculate 
      # within stage cumulative times 
        # For these cases: observed event, left censor, right censor, and earliest 
        # of the pair of interval censor times
          for(k in 1:K) {
            # Logical tests
              test_gt_k[i,k] <- ifelse(times[i] > a_jags[i,k],1,0) # time>cutpoint k
              test_ltet_kplus1[i,k] <- ifelse(times[i] <= a_jags[i,k+1],1,0) # time<=k+1
              test_gt_kplus1[i,k] <- ifelse(times[i] > a_jags[i,k+1],1,0) # time>cutpoint k+1
            # Time within this stage? 1 = yes, 0 = no
              int_obs_m[i,k] <- test_gt_k[i,k]*test_ltet_kplus1[i,k]
            # Record time within stage.  time[i] w/in stage => cumulative time, else
            # time > end of this stage => total stage time, else record a 0.
              R[i,k] <- ifelse(int_obs_m[i,k]==1,
                    (times[i]-a_jags[i,k]),test_gt_kplus1[i,k]*(a_jags[i,k+1]-a_jags[i,k]))
          }
          # Track the stage number in which time[i] falls
            int_obs_v[i] <- inprod(int_obs_m[i,1:K] , 1:K) 
        # For these cases: latter of the pair of interval censor times
          for(k in 1:K) {
              test_gt_k_2[i,k] <- ifelse(times_2[i] > a_jags[i,k],1,0)
              test_ltet_kplus1_2[i,k] <- ifelse(times_2[i] <= a_jags[i,k+1],1,0)
              test_gt_kplus1_2[i,k] <- ifelse(times_2[i] > a_jags[i,k+1],1,0)
              int_obs_m_2[i,k] <- test_gt_k_2[i,k]*test_ltet_kplus1_2[i,k]
              R_2[i,k] <- ifelse(int_obs_m_2[i,k]==1,
                    (times_2[i]-a_jags[i,k]),test_gt_kplus1_2[i,k]*(a_jags[i,k+1]-a_jags[i,k]))
          }
      # Populate pieces of the cumulative hazard by stage
        for(k in 1:K){
          # For observed event, left-, right-, and earlier of interval censor times
            haz_m[i,k] <- lambda[k]*R[i,k] 
          # Only for the latter of pair of censor times for an interval censor event
            haz_m_2[i,k] <- lambda[k]*R_2[i,k] 
        }
      # Cumulative hazard functions
        H[i] <- sum(haz_m[i,1:K])
        H_2[i] <- sum(haz_m_2[i,1:K])
    }
    
  # Likelihood
    for(i in 1:n){
      # Log hazard function: proportional hazards effects lam_k*exp(X_k'B_k)
        log_h[i] <- log(lambda[int_obs_v[i]]) 
      # Log survival function: S=exp(-H(t))
        log_S[i] <- -H[i] 
      # Log of (1-survival) function
        log_minus_S[i] <- log(1-exp(-H[i]))
      # Log of S_time1 (lower) minus log of S_time2 (upper) for an interval censor event 
      # ifelse() to avoid log(0) when an event is NOT an interval censor, i.e. time_2=0
        diff_S1_S2[i] <- ifelse(H_2[i]>0,exp(-H[i]) - exp(-H_2[i]), 1) 
        log_diff_S1_S2[i] <- log( diff_S1_S2[i] ) # Returns log(1)=0 if time_2=0
      # The log likelihood and zeros trick. Recall, indicators=1 if censorship occurs, 0 otherwise
        log_like[i] <- ( 
                # Observed event, f(t)=h(t)S(t), all censor indicators = 0
                  (1-delta[i])*(1-omega[i])*(1-rho[i])*(log_h[i]+log_S[i])
                # Right censor (delta=1), survival up to right censor time 
                  + delta[i]*(1-omega[i])*(1-rho[i])*log_S[i]          
                # Left censor (omega=1), cumulative mortality (1-S) to left censor time
                  + omega[i]*(1-delta[i])*(1-rho[i])*log_minus_S[i]      
                # Interval censor (rho=1), S to lower minus S to upper interval times 
                  + rho[i]*(1-delta[i])*(1-omega[i])*(log_diff_S1_S2[i]) 
                )  
        phi[i] <- C_pois - log_like[i] # Add a constant to ensure phi is positive
        zeros[i] ~ dpois(phi[i]) # Zeros vector passed to JAGS
    }
  
  # Derived par. for deviance, e.g. useful for pV=var(deviance[])/2) DIC complexity term  
    deviance <- -2*sum(log_like[1:n])  
} # end model
", file="TwoStageSurvivalModelNoCovsStage1LengthEstimated.txt")
```

Code to package data and fit the model.

```
# User specifies stages, any fixed cutpoints, and priors ------------------------- 
  # Time interval cutpoints
    m <- 2 # Number of survival stages (subsequently, m+1 cutpoints)
    a1 <- 0 # Fix the first interval cutpoint at 0
    # a2 The time spent in (and thus the exit time out of) the first stage is estimated
    a3 <- 999 # Third and final cutpoint, set suitably large to exceed the maximum observed time 
  # Prior specifications
    # Hazard rates
      # Baseline hazards: vague uniform priors
        lambda_L.v <- c(0.0001, 0.0001)
        lambda_U.v <- c(2.0, 2.0)      
    # Time spent in the first survival stage
      # Baseline: vague uniform prior
        tran_time_prior_L.v <- c(2)
        tran_time_prior_U.v <- c(100)
        
  # JAGS model fit specifications (low sample iterations for demonstration purposes)    
    n_chains <- 3
    n_adapt <- 100
    n_update_iter <- 500
    n_sample_iter <- 500
    n_sample_thin <- 2
  # Specify JAGS nodes to store  
    p.jags <- c(
    # Estimated parameters (initial values needed)
      "lambda","tran_time_base",
    # Derived parameters (no initial values needed)
      "deviance")
  # Specify initial values for estimated parameters
    i.jags <- function() {list(lambda = c(0.8,0.01) * runif(n=2,min=0.9,max=1.1),
        tran_time_base = 10 + runif(1,-2,2)
        )}         
# End user specifies ----------------------------------------------------------

# Packaging for JAGS runs
  # Event time data
    times <- data.df$Time
    times_2 <- data.df$Time_2
    delta <- data.df$Delta
    omega <- data.df$Omega  
    rho <- data.df$Rho
  # Create a list for JAGS
    d.jags <- list( 
      # Data and survival stage structure parameters
        times = times, times_2 = times_2, delta = delta, omega = omega, rho = rho,
        K = m, n = length(times), a1 = a1, a3 = a3, 
      # Priors and related parameter dimensions       
        lambda_L.v = lambda_L.v, lambda_U.v = lambda_U.v, 
        tran_time_prior_L.v = tran_time_prior_L.v, tran_time_prior_U.v = tran_time_prior_U.v,  
      # Placeholder zeros and a constant 'C_pois' for the zeros likelihood 
      # specification trick
        zeros = rep(0, length(times)), C_pois = 100000
      ) # End d.jags
      
# Fit the JAGS model
  mod <- jags.model(data = d.jags, 
        file = "TwoStageSurvivalModelNoCovsStage1LengthEstimated.txt",
        inits = i.jags, n.chains = n_chains, n.adapt = n_adapt)
  update(mod, n_update_iter)
  mcmc_samples <- coda.samples(mod, variable.names = p.jags, n.iter = n_sample_iter, thin = n_sample_thin)

# Explore convergence, posterior summaries, and DIC
  gelman.diag(mcmc_samples) # R hat statistics
  summary(mcmc_samples)
  dev.v <- as.mcmc(do.call(rbind,mcmc_samples))[,"deviance"]
  mean(dev.v)+var(dev.v)/2 # DIC using 'pV' penalty term
```

*Model G: 1-stage model, covariate
effects on survival. This model is equivalent to a standard exponential
survival model with covariate effects.*

```
# Model script
cat("
model{
  # Priors
    # Baseline hazard rates
      for(k in 1:K){
        lambda[k]~dunif(lambda_L.v[k],lambda_U.v[k])
      }
    # Coefficients in linear predictors for baseline hazard rates  
      for(p in 1:Nbetas1){
        beta1[p]~dunif(coeff_lambda1_L.v[p],coeff_lambda1_U.v[p])
      }
      
  # Assemble components for the likelihood
    for(i in 1:n){
      # Define time interval cutpoints, here fixed cutpoints are passed to JAGS
        a_jags[i,1:(K+1)] <- c(a1,a2) 
      # Determine in which stage a given event time falls and calculate 
      # within stage cumulative times 
        # For these cases: observed event, left censor, right censor, and earliest 
        # of the pair of interval censor times
          for(k in 1:K) {
            # Logical tests
              test_gt_k[i,k] <- ifelse(times[i] > a_jags[i,k],1,0) # time>cutpoint k
              test_ltet_kplus1[i,k] <- ifelse(times[i] <= a_jags[i,k+1],1,0) # time<=k+1
              test_gt_kplus1[i,k] <- ifelse(times[i] > a_jags[i,k+1],1,0) # time>cutpoint k+1
            # Time within this stage? 1 = yes, 0 = no
              int_obs_m[i,k] <- test_gt_k[i,k]*test_ltet_kplus1[i,k]
            # Record time within stage.  time[i] w/in stage => cumulative time, else
            # time > end of this stage => total stage time, else record a 0.
              R[i,k] <- ifelse(int_obs_m[i,k]==1,
                    (times[i]-a_jags[i,k]),test_gt_kplus1[i,k]*(a_jags[i,k+1]-a_jags[i,k]))
          }
          # Track the stage number in which time[i] falls
            int_obs_v[i] <- inprod(int_obs_m[i,1:K] , 1:K) 
        # For these cases: latter of the pair of interval censor times
          for(k in 1:K) {
              test_gt_k_2[i,k] <- ifelse(times_2[i] > a_jags[i,k],1,0)
              test_ltet_kplus1_2[i,k] <- ifelse(times_2[i] <= a_jags[i,k+1],1,0)
              test_gt_kplus1_2[i,k] <- ifelse(times_2[i] > a_jags[i,k+1],1,0)
              int_obs_m_2[i,k] <- test_gt_k_2[i,k]*test_ltet_kplus1_2[i,k]
              R_2[i,k] <- ifelse(int_obs_m_2[i,k]==1,
                    (times_2[i]-a_jags[i,k]),test_gt_kplus1_2[i,k]*(a_jags[i,k+1]-a_jags[i,k]))
          }
      # Prepopulate interval-specific linear predictors for lambdas
        elinpred[i,1] <- exp(inprod(beta1[],X1[i,])) 
      # Populate pieces of the cumulative hazard by stage
        for(k in 1:K){
          # For observed event, left-, right-, and earlier of interval censor times
            haz_m[i,k] <- elinpred[i,k]*lambda[k]*R[i,k] 
          # Only for the latter of pair of censor times for an interval censor event
            haz_m_2[i,k] <- elinpred[i,k]*lambda[k]*R_2[i,k] 
        }
      # Cumulative hazard functions
        H[i] <- sum(haz_m[i,1:K])
        H_2[i] <- sum(haz_m_2[i,1:K])
    }
    
  # Likelihood
    for(i in 1:n){
      # Log hazard function: proportional hazards effects lam_k*exp(X_k'B_k)
        log_h[i] <- log(lambda[int_obs_v[i]]*elinpred[i,int_obs_v[i]]) 
      # Log survival function: S=exp(-H(t))
        log_S[i] <- -H[i] 
      # Log of (1-survival) function
        log_minus_S[i] <- log(1-exp(-H[i]))
      # Log of S_time1 (lower) minus log of S_time2 (upper) for an interval censor event 
      # ifelse() to avoid log(0) when an event is NOT an interval censor, i.e. time_2=0
        diff_S1_S2[i] <- ifelse(H_2[i]>0,exp(-H[i]) - exp(-H_2[i]), 1) 
        log_diff_S1_S2[i] <- log( diff_S1_S2[i] ) # Returns log(1)=0 if time_2=0
      # The log likelihood and zeros trick. Recall, indicators=1 if censorship occurs, 0 otherwise
        log_like[i] <- ( 
                # Observed event, f(t)=h(t)S(t), all censor indicators = 0
                  (1-delta[i])*(1-omega[i])*(1-rho[i])*(log_h[i]+log_S[i])
                # Right censor (delta=1), survival up to right censor time 
                  + delta[i]*(1-omega[i])*(1-rho[i])*log_S[i]          
                # Left censor (omega=1), cumulative mortality (1-S) to left censor time
                  + omega[i]*(1-delta[i])*(1-rho[i])*log_minus_S[i]      
                # Interval censor (rho=1), S to lower minus S to upper interval times 
                  + rho[i]*(1-delta[i])*(1-omega[i])*(log_diff_S1_S2[i]) 
                )  
        phi[i] <- C_pois - log_like[i] # Add a constant to ensure phi is positive
        zeros[i] ~ dpois(phi[i]) # Zeros vector passed to JAGS
    }
  
  # Derived par. for deviance, e.g. useful for pV=var(deviance[])/2) DIC complexity term  
    deviance <- -2*sum(log_like[1:n])  
} # end model
", file="OneStageSurvivalModelWithCovs.txt")
```

Code to package data and fit the model.

```
# User specifies stages, any fixed cutpoints, and priors ------------------------- 
  # Time interval cutpoints
    m <- 1 # Number of survival stages (subsequently, m+1 cutpoints)
    a1 <- 0 # Fix the first interval cutpoint at 0
    a2 <- 999 # Second and final cutpoint, set suitably large to exceed the maximum observed time
  # Covariate matrices
    X1 <- model.matrix(~-1+Size,data=data.df)
  # Prior specifications
    # Hazard rates
      # Baseline hazards: vague uniform priors
        lambda_L.v <- c(0.0001)
        lambda_U.v <- c(2.0)    
      # Beta's for covariate effects on lambdas: vague uniform priors
        coeff_lambda1_L.v <- c(-0.25)
        coeff_lambda1_U.v <- c(0.25)        
  # JAGS model fit specifications (low sample iterations for demonstration purposes)     
    n_chains <- 3
    n_adapt <- 100
    n_update_iter <- 500
    n_sample_iter <- 500
    n_sample_thin <- 2
  # Specify JAGS nodes to store  
    p.jags <- c(
    # Estimated parameters (initial values needed)
      "lambda","beta1",
    # Derived parameters (no initial values needed)
      "deviance")
  # Specify initial values for estimated parameters
    i.jags <- function() {list(lambda = 0.1 * runif(n=1,min=0.9,max=1.1),
        beta1 = 0 + runif(1,-0.005,0.005) 
        )}         
# End user specifies ----------------------------------------------------------

# Packaging for JAGS runs
  # Event time data
    times <- data.df$Time
    times_2 <- data.df$Time_2
    delta <- data.df$Delta
    omega <- data.df$Omega
    rho <- data.df$Rho  
  # Create a list for JAGS
    d.jags <- list( 
      # Data and survival stage structure parameters
        times = times, times_2 = times_2, delta = delta, omega = omega, rho = rho,
        K = m, n = length(times), a1 = a1, a2 = a2, X1 = X1, 
      # Priors and related parameter dimensions       
        lambda_L.v = lambda_L.v, lambda_U.v = lambda_U.v, 
        coeff_lambda1_L.v = coeff_lambda1_L.v, coeff_lambda1_U.v = coeff_lambda1_U.v,
        # Numbers of estimated coefficients
        Nbetas1 = ncol(X1),
      # Placeholder zeros and a constant 'C_pois' for the zeros likelihood 
      # specification trick
        zeros = rep(0, length(times)), C_pois = 100000
      ) # End d.jags
      
# Fit the JAGS model
  mod <- jags.model(data = d.jags, 
        file = "OneStageSurvivalModelWithCovs.txt",
        inits = i.jags, n.chains = n_chains, n.adapt = n_adapt)
  update(mod, n_update_iter) # Burn in
  mcmc_samples <- coda.samples(mod, variable.names = p.jags, n.iter = n_sample_iter, thin = n_sample_thin)

# Explore convergence, posterior summaries, and DIC
  gelman.diag(mcmc_samples) # R hat statistics
  summary(mcmc_samples)
  dev.v <- as.mcmc(do.call(rbind,mcmc_samples))[,"deviance"]
  mean(dev.v)+var(dev.v)/2 # DIC using 'pV' penalty term
```

*Model H: 1-stage model, but no
covariate effects on survival. This model is equivalent to a standard
exponential survival model without covariate effects.*

```
# Model script
cat("
model{
  # Priors
    # Baseline hazard rates
      for(k in 1:K){
        lambda[k]~dunif(lambda_L.v[k],lambda_U.v[k])
      }
      
  # Assemble components for the likelihood
    for(i in 1:n){
      # Define time interval cutpoints, here fixed cutpoints are passed to JAGS
        a_jags[i,1:(K+1)] <- c(a1,a2) 
      # Determine in which stage a given event time falls and calculate 
      # within stage cumulative times 
        # For these cases: observed event, left censor, right censor, and earliest 
        # of the pair of interval censor times
          for(k in 1:K) {
            # Logical tests
              test_gt_k[i,k] <- ifelse(times[i] > a_jags[i,k],1,0) # time>cutpoint k
              test_ltet_kplus1[i,k] <- ifelse(times[i] <= a_jags[i,k+1],1,0) # time<=k+1
              test_gt_kplus1[i,k] <- ifelse(times[i] > a_jags[i,k+1],1,0) # time>cutpoint k+1
            # Time within this stage? 1 = yes, 0 = no
              int_obs_m[i,k] <- test_gt_k[i,k]*test_ltet_kplus1[i,k]
            # Record time within stage.  time[i] w/in stage => cumulative time, else
            # time > end of this stage => total stage time, else record a 0.
              R[i,k] <- ifelse(int_obs_m[i,k]==1,
                    (times[i]-a_jags[i,k]),test_gt_kplus1[i,k]*(a_jags[i,k+1]-a_jags[i,k]))
          }
          # Track the stage number in which time[i] falls
            int_obs_v[i] <- inprod(int_obs_m[i,1:K] , 1:K) 
        # For these cases: latter of the pair of interval censor times
          for(k in 1:K) {
              test_gt_k_2[i,k] <- ifelse(times_2[i] > a_jags[i,k],1,0)
              test_ltet_kplus1_2[i,k] <- ifelse(times_2[i] <= a_jags[i,k+1],1,0)
              test_gt_kplus1_2[i,k] <- ifelse(times_2[i] > a_jags[i,k+1],1,0)
              int_obs_m_2[i,k] <- test_gt_k_2[i,k]*test_ltet_kplus1_2[i,k]
              R_2[i,k] <- ifelse(int_obs_m_2[i,k]==1,
                    (times_2[i]-a_jags[i,k]),test_gt_kplus1_2[i,k]*(a_jags[i,k+1]-a_jags[i,k]))
          }
      # Populate pieces of the cumulative hazard by stage
        for(k in 1:K){
          # For observed event, left-, right-, and earlier of interval censor times
            haz_m[i,k] <- lambda[k]*R[i,k] 
          # Only for the latter of pair of censor times for an interval censor event
            haz_m_2[i,k] <- lambda[k]*R_2[i,k] 
        }
      # Cumulative hazard functions
        H[i] <- sum(haz_m[i,1:K])
        H_2[i] <- sum(haz_m_2[i,1:K])
    }
    
  # Likelihood
    for(i in 1:n){
      # Log hazard function
        log_h[i] <- log(lambda[int_obs_v[i]]) 
      # Log survival function: S=exp(-H(t))
        log_S[i] <- -H[i] 
      # Log of (1-survival) function
        log_minus_S[i] <- log(1-exp(-H[i]))
      # Log of S_time1 (lower) minus log of S_time2 (upper) for an interval censor event 
      # ifelse() to avoid log(0) when an event is NOT an interval censor, i.e. time_2=0
        diff_S1_S2[i] <- ifelse(H_2[i]>0,exp(-H[i]) - exp(-H_2[i]), 1) 
        log_diff_S1_S2[i] <- log( diff_S1_S2[i] ) # Returns log(1)=0 if time_2=0
      # The log likelihood and zeros trick. Recall, indicators=1 if censorship occurs, 0 otherwise
        log_like[i] <- ( 
                # Observed event, f(t)=h(t)S(t), all censor indicators = 0
                  (1-delta[i])*(1-omega[i])*(1-rho[i])*(log_h[i]+log_S[i])
                # Right censor (delta=1), survival up to right censor time 
                  + delta[i]*(1-omega[i])*(1-rho[i])*log_S[i]          
                # Left censor (omega=1), cumulative mortality (1-S) to left censor time
                  + omega[i]*(1-delta[i])*(1-rho[i])*log_minus_S[i]      
                # Interval censor (rho=1), S to lower minus S to upper interval times 
                  + rho[i]*(1-delta[i])*(1-omega[i])*(log_diff_S1_S2[i]) 
                )  
        phi[i] <- C_pois - log_like[i] # Add a constant to ensure phi is positive
        zeros[i] ~ dpois(phi[i]) # Zeros vector passed to JAGS
    }
  
  # Derived par. for deviance, e.g. useful for pV=var(deviance[])/2) DIC complexity term  
    deviance <- -2*sum(log_like[1:n])  
} # end model
", file="OneStageSurvivalModelNoCovs.txt")
```

Code to package data and fit the model.

```
# User specifies stages, any fixed cutpoints, and priors ------------------------- 
  # Time interval cutpoints
    m <- 1 # Number of survival stages (subsequently, m+1 cutpoints)
    a1 <- 0 # Fix the first interval cutpoint at 0
    a2 <- 999 # Second and cutpoint, set suitably large to exceed the maximum observed time
  # Prior specifications
    # Hazard rates
      # Baseline hazards: vague uniform priors
        lambda_L.v <- c(0.0001)
        lambda_U.v <- c(2.0)        
  # JAGS model fit specifications (low sample iterations for demonstration purposes)    
    n_chains <- 3
    n_adapt <- 100
    n_update_iter <- 250
    n_sample_iter <- 250
    n_sample_thin <- 2
  # Specify JAGS nodes to store  
    p.jags <- c(
    # Estimated parameters (initial values needed)
      "lambda",
    # Derived parameters (no initial values needed)
      "deviance")
  # Specify initial values for estimated parameters
    i.jags <- function() {list(lambda = 0.1 * runif(n=1,min=0.9,max=1.1) 
        )}         
# End user specifies ----------------------------------------------------------

# Packaging for JAGS runs
  # Event time data
    times <- data.df$Time
    times_2 <- data.df$Time_2
    delta <- data.df$Delta  
    omega <- data.df$Omega  
    rho <- data.df$Rho  
  # Create a list for JAGS
    d.jags <- list( 
      # Data and survival stage structure parameters
        times = times, times_2 = times_2, delta = delta, omega = omega, rho = rho,
        K = m, n = length(times), a1 = a1, a2 = a2,  
      # Priors and related parameter dimensions       
        lambda_L.v = lambda_L.v, lambda_U.v = lambda_U.v,
      # Placeholder zeros and a constant 'C_pois' for the zeros likelihood 
      # specification trick
        zeros = rep(0, length(times)), C_pois = 100000
      ) # End d.jags
      
# Fit the JAGS model
  mod <- jags.model(data = d.jags, 
        file = "OneStageSurvivalModelNoCovs.txt",
        inits = i.jags, n.chains = n_chains, n.adapt = n_adapt)
  update(mod, n_update_iter)
  mcmc_samples <- coda.samples(mod, variable.names = p.jags, n.iter = n_sample_iter, thin = n_sample_thin)

# Explore convergence, posterior summaries, and DIC
  gelman.diag(mcmc_samples) # R hat statistics
  summary(mcmc_samples)
  dev.v <- as.mcmc(do.call(rbind,mcmc_samples))[,"deviance"]
  mean(dev.v)+var(dev.v)/2 # DIC using 'pV' penalty term
```

### **Supplemental Figures**

**Figure S1.** Covariate effects of a simulated
“specimen size” covariate effect on stage-specific survival and the time
spent in the second “acclimation” stage of a 3-stage time-to-event
survival process. Size values are simulated to range from 1 to 100
generic units, and vertical and horizontal line segments show the
resultant survival and time spent in the acclimation (stage 2) interval
for the median sized individual.

**Figure S2.** Bias simulation testing results for a
3-stage time-to-event survival model without covariate effects on
baseline parameters. Gray points indicate percent error (100\*(estimated
parameter value – true parameter value) / true parameter value) results
for each respective data set simulation and estimation iteration. Blue
lines indicate median percent error values across simulations (50 data
sets simulated for each sample size). Lambda’s represent stage-specific
baseline hazard rates and “transition time” represents the baseline time
spent in the second survival stage.

**Figure S3.** Bias simulation testing results for a
3-stage time-to-event survival model without covariate effects on
baseline parameters and with the addition of interval censorship events
(models also include left censorship in the form of deaths in stage 1
and right censorship in the form of terminal censorship events for
full-term survival specimens). Simulations include a 25 d interval
censorship window in the second survival stage and a 100 d interval
censorship in the third survival stage, with a target interval
censorship rate of 10% of subjects. Gray points indicate percent error
(100\*(estimated parameter value – true parameter value) / true parameter
value) results for each respective data set simulation and estimation
iteration. Blue lines indicate median percent error values across
simulations (50 data sets simulated for each sample size). Lambda’s
represent stage-specific baseline hazard rates and “transition time”
represents the baseline time spent in the second survival stage.

**Figure S4.** Parameter estimate precision outcomes
from simulation testing of 3-stage time-to-event survival models with
(top two rows, blue lines) and without (bottom row, yellow lines)
covariate effects on baseline parameters. Precision is presented as the
percent coefficient of variation based on posterior samples for a given
model fit and estimated parameter (i.e. 100 \* standard deviation of
posterior samples / mean of posterior samples). Gray points indicate
percent CV results for each respective data set simulation and
estimation iteration. Lines indicate median CV values across simulations
(50 data sets simulated for each sample size). Lambda’s represent
stage-specific baseline hazard rates and “transition time” represents
the baseline time spent in the second survival stage. If specified in a
given model, beta’s are coefficients on the linear predictor scale for
covariate effects on hazard rates and zeta is the coefficient on the
linear predictor scale for covariate effects on the time in stage
two.

**Figures S5.**
Estimated survival curves for the best DIC-supported case study
multistage models for cisco (left panel) and wild turkey (right panel)
plotted against empirical Kaplan-Meier curves. For cisco, the best
DIC-supported multistage model included three survival stages with age
effects on all stage-specific survivals and the time spent in the second
“acclimation” stage. For wild turkey, the best DIC-supported multistage
model included two stages with release weight effects on stage one
survival only. Separate Kaplan-Meier curves are provided for the Age 0
and Age 1 groups for cisco. A single Kaplan-Meier curve encompassing all
birds is provided for wild turkey and compared against the estimated
multistage survival for the 50th percentile release weight bird.
